# Mycobacterial PIMs Inhibit Host Inflammatory Responses through CD14-Dependent and CD14-Independent Mechanisms

**DOI:** 10.1371/journal.pone.0024631

**Published:** 2011-09-16

**Authors:** Nathalie Court, Stéphanie Rose, Marie-Laure Bourigault, Sophie Front, Olivier R. Martin, Jennifer K. Dowling, Elaine F. Kenny, Luke O'Neill, François Erard, Valerie F. J. Quesniaux

**Affiliations:** 1 University of Orléans Molecular Immunology and Embryology, Orléans, France; 2 CNRS UMR6218, Orléans, France; 3 University of Orléans Institut de Chimie Organique et Analytique, Orléans, France; 4 CNRS UMR6005, Orléans, France; 5 School of Biochemistry and Immunology, Trinity College Dublin, Ireland; University of California Los Angeles, United States of America

## Abstract

Mycobacteria develop strategies to evade the host immune system. Among them, mycobacterial LAM or PIMs inhibit the expression of pro-inflammatory cytokines by activated macrophages. Here, using synthetic PIM analogues, we analyzed the mode of action of PIM anti-inflammatory effects. Synthetic PIM_1_ isomer and PIM_2_ mimetic potently inhibit TNF and IL-12 p40 expression induced by TLR2 or TLR4 pathways, but not by TLR9, in murine macrophages. We show inhibition of LPS binding to TLR4/MD2/CD14 expressing HEK cells by PIM_1_ and PIM_2_ analogues. More specifically, the binding of LPS to CD14 was inhibited by PIM_1_ and PIM_2_ analogues. CD14 was dispensable for PIM_1_ and PIM_2_ analogues functional inhibition of TLR2 agonists induced TNF, as shown in CD14-deficient macrophages. The use of rough-LPS, that stimulates TLR4 pathway independently of CD14, allowed to discriminate between CD14-dependent and CD14-independent anti-inflammatory effects of PIMs on LPS-induced macrophage responses. PIM_1_ and PIM_2_ analogues inhibited LPS-induced TNF release by a CD14-dependent pathway, while IL-12 p40 inhibition was CD14-independent, suggesting that PIMs have multifold inhibitory effects on the TLR4 signalling pathway.

## Introduction

Mycobacterium tuberculosis induces the formation of granuloma, a “super cellular” structure involving cells both from the innate and the adaptive immune responses, that may play a dual role, for infection containment on the one side, and immune evasion and persistence of viable mycobacteria on the other side. *M. tuberculosis* are recognized by multiple pattern recognition receptors expressed on alveolar macrophages, their primary host cells, which in turn modulate the immune responses by secreting cytokines and chemokines. TNF, an essential mediator for granuloma formation, is essential for controlling *M. tuberculosis* infection [1], [2], together with IL-12, IFNγ or IL-1 [3]–[9]. Macrophages also express cytokines that dampen the immune response such as IL-10. Mycobacteria produce a series of molecules modulating the immune system, including the protein ESAT-6, lipomannans (LM), mannose-capped lipoarabinomannan (ManLAM) and their precursors mono- to tetra-acylated phosphatidyl-*myo*-inositol mannosides (PIM; lyso-PIM for one acyl, PIM for two acyl, Ac_1_PIM for three acyl and Ac_2_PIM for four acyl, respectively) [10]–[19].

Several pattern recognition receptors have been implicated in the recognition of mycobacterial LAM, LM and PIMs by macrophages and dendritic cells, such as Toll-like receptors (TLRs) and C-type lectin mannose receptor (CD206) and dendritic cell-specific intercellular adhesion molecule-3 grabbing nonintegrin (DC-SIGN/CD209) [15]–[17], [20]–[28]. Tri and tetra-acylated LM fractions are pro-inflammatory through TLR2, TLR4 and myeloid differentiation protein 88 (MyD88), and purified fractions of dimannoside PIM_2_ and hexamannoside PIM_6_, the two most abundant classes of PIMs found in *M. tuberculosis* H37Rv and *M. bovis* BCG (bacillus Calmette Guérin), may be proinflammatory through TLR2 [29], [30]. Higher-order PIMs with mannose cap-like structures seem to associate with human mannose receptor and to contribute to phagosome-lysosome fusion depending of their degree of acylation, while PIM_2_ are recognized by DC-SIGN independently of their acylation degree [31].

Among the anti-inflammatory activities, ManLAM inhibition of LPS-induced IL-12 production in dendritic cells was attributed to DC-SIGN [15]. We showed recently that di-acylated LM, but also purified fractions of PIM_2_ and PIM_6,_ and synthetic PIM_1_ and PIM_2_ analogues inhibit LPS/TLR4-induced cytokine response independently of TLR2, SIGN-R1 and mannose receptor [18], [19]. Suppression of ovalbumin-induced allergic airway eosinophilia, a model dependent on LPS response [32], by natural or synthetic PIMs, and by a PIM_2_ analogue was reported [33]–[35]. Thus, not only complex mycobacterial lipoglycans like ManLAM and LM, but also small molecular weight PIMs are potent inhibitors of host inflammatory responses.

LAM were also shown to insert into mononuclear cell plasma membranes [36] and to modify the signalling machineries of rafts/microdomains [37]. LAM GPI anchor PIM_6_ competitively inhibited LAM insertion into plasma membranes, likely into specialized domains enriched in endogenous GPI-anchored molecules [36]. Although TLR4 is a major receptor for the cellular response to LPS, cells need to express co-receptors such as the GPI-anchored CD14 or MD2 to mount a full response to LPS. MD2 is indeed necessary for the processing and membrane expression of TLR4 as well as for LPS signalling [38]–[40] while CD14 is required for the LPS binding to MD2/TLR4 and subsequent signalling [41], [42].

Here, using synthetic PIM_1_ and PIM_2_ analogues, we analyzed the mode of action of PIM anti-inflammatory effects. We investigated LPS binding on TLR4/MD2/CD14 expressing cells and found that PIMs inhibit this step and more specifically the LPS binding to CD14. By using a shorter form of LPS, rough-LPS, that stimulates TLR4 pathway independently of CD14 [41], we then discriminated between CD14-dependent and CD14-independent anti-inflammatory effects of PIMs on the LPS-induced response. Our data show that PIM_1_ and PIM_2_ analogues inhibit the LPS-induced TNF production by a CD14-dependent pathway while the IL-12 p40 inhibition is CD14-independent, suggesting that PIMs have multifold inhibitory effects on TLR4 signalling pathway.

## Materials and Methods

### Ethics statement

The study of immune responses to mycobacteria infections was approved by the Regional ethics committee for animal experiments (CL2008-011).

### Mice

Six to 12 week old mice deficient for TLR2 [43], TLR4 [44], CD14 (obtained from Freeman, M.W [45]), MD2 [39] and wild-type C57Bl/6 mice were bred at the Transgenose Institute animal facility (UPS44 TAAM, Orleans, France).

### Synthetic PIMs

PIM_1_ containing a C_16_ and a C_18_ chain in the glycerolipid unit (2-*O*-α-D-mannopyranosyl-1-*O*-phosphatidyl-D-*myo*-inositol), together with the PIM_1_ isomer (1-*O*-α-D-mannopyranosyl-2-*O*-phosphatidyl-D-*myo*-inositol), PIM_2_ mimetic [1,3-bis-(α-D-manno pyranosyl)-2-*O*-phosphatidyl glycerol], and the reference compound phosphatidyl inositol (PI, 1-*O*-phosphatidyl-D-*myo*-inositol), were prepared as described [19].

### LPS binding to cells

Human embryonic kidney (HEK) 293 cells were obtained from the Centre for Applied Microbiology and Research (Porton Down, Salisbury, Wiltshire, UK) and were maintained in Dulbecco's modified Eagle's medium (DMEM) supplemented with 10% fetal calf serum, 100 U/mL penicillin, 100 µg/mL streptomycin and 2 mm L-glutamine and maintained at 37°C in a humidified atmosphere of 5% CO_2_. HEK293 cells transfected with TLR4/MD2/CD14 (HEK-MTC) were obtained from InvivoGen (San Diego, CA) and maintained in the same medium as above supplemented with Hygrogold (InvivoGen) and blastocidin (InvivoGen). HEK-MTC cells (1×10^6^ cells in 50 µl in DMEM 10% FCS) were incubated with 10 µg/mL of PIM or vehicle for 30 min at 37°C under gentle agitation prior incubation with biotinylated smooth LPS (S-LPS; *Escherichia coli*, serotype O111:B4, InvivoGen) at a final concentration of 2.5 µg/mL prepared in DMEM 10% FCS for 15–20 min. Cells were washed with ice cold PBS and stained with streptavidin-FITC on ice. After fixation with 3% paraformaldehyde, binding of S-LPS-biotin to cells was measured on a BD FACS Calibur™. S-LPS-binding on bone marrow derived macrophages (see below) was also investigated by using DMEM supplemented with 0.1% FCS and a final concentration of 5 µg/mL of S-LPS-biotin prepared in DMEM 0.1% FCS and S-LPS-binding was measured with a BD FACS Canto™ II.

### LPS binding to soluble CD14

Soluble recombinant mouse CD14 was coated overnight at 4°C (5 µg/mL on Nunc 96-well plates; R&D systems, Abingdon, UK) and non specific binding saturated with 2% BSA in PBS for 1 hr at 37°C. The plates were washed three times in PBS before incubation with synthetic PIMs (10 µg/mL; 1 hr at 37°C) before addition of biotinylated S-LPS for 2 hrs at 37°C (100 ng/mL, InvivoGen) in PBS containing 1% of fetal calf serum. Alternatively, 0.1% serum from wild-type or LBP-deficient mice [46] was used, as indicated. Unbound S-LPS-biotin was removed with four PBS washes, and bound S-LPS-biotin was detected with horseradish peroxidase avidin D conjugate (1/2000, Vector laboratories) diluted in 1% BSA in PBS. After addition of the ABTS substrate (2,2′-azino-bis-(3-ethylbenzthiazoline-6-sulfonic acid at 0.3 g/L in 0.1 M anhydrous citric acid containing 0.3% H_2_O_2_), absorbance at 405 nm was measured with a microplate reader (Bio-Tek Instrument, INC). Competition with increasing concentrations of ultrapure S-LPS (*E. coli*, serotype O111:B4, InvivoGen, San Diego, CA) was performed to assess binding specificity.

### Cell culture

Murine bone marrow cells were isolated from femurs and cultivated (10^6^/mL) for 7 days in DMEM supplemented with 2 mM L-glutamine, 20% horse serum and 30% L929 cell-conditioned medium as source of M-CSF. After further three days in fresh medium, the cell preparation contained a homogenous population of macrophages (97–98% CD11b^+^F4/80^+^). The bone marrow derived macrophages (BMDM; 10^5^ cells/well) in DMEM supplemented with 2 mM L-glutamine and 0.1% FCS were stimulated with 100 ng/mL of S-LPS (*E. coli*, serotype O111:B4, CD14-dependent, ultrapure S-LPS from InvivoGen or Sigma, St Louis, MO), 0.5 µg/mL of synthetic bacterial lipopeptide Pam_3_CSK_4_ ([S-[2,3-bis-(palmitoyloxy)-(2-RS)-propyl]-N-palmitoyl-(R)-Cys-(S)-Ser-Lys_4_-OH], tri hydro-chloride, EMC Microcollections, Tuebingen, Germany), 30 ng/mL Malp2 (S-(2,3-bis Acyloxypropyl)-cysteine-GNNDESNISFKEK, Alexis Biochemicals, Lausanne, Switzerland), 0.125 µM of CpG ODN1826 (tccatgacgttcctgacgtt, Invivogen), 3 µM Taxol (Alexis). A CD14-independent, rough-LPS (Re-LPS; *E. coli*, serotype 515, Alexis) was also used, as indicated. Lyophilised PIM preparations were solubilised in DMSO and added to the cultures at the indicated concentration 30 min prior to the stimuli in a solution containing a maximum non-cytotoxic, 1% DMSO final concentration. Vehicle controls at the relevant DMSO concentration are included in each experiment. PIMs were incubated in presence or absence of 5 µg/mL recombinant mouse CD14 Fc chimera (endotoxin <1.0EU/µg protein; R&D system). The macrophages were activated with IFN-γ (500 U/mL) to study IL-12 release, and the supernatants harvested after 24 hours for further analysis. Absence of cytotoxicity of the stimuli was controlled using MTT incorporation. To control PIMs anti-inflammatory activity on human embryonic kidney (HEK) cells, HEK-MTC cells (6×10^4^ cells/well) were stimulated with S-LPS in the presence of PIM analogues as above and human IL-8 concentration was measured in the supernatant after overnight incubation.

### Cytokine ELISA

Supernatants were harvested and assayed for cytokine content using commercially available ELISA reagents for murine TNF, murine IL-12 p40, and human IL-8 (Duoset R&D Systems).

### Statistical analysis

Statistical significance was determined with Graph Pad Prism software (version 4.0, San Diego, CA) by one or two way parametric ANOVA test followed by Bonferroni post-test. P values<0.05 were considered statistically significant.

## Results

### Interference of PIMs with LPS binding to cells

**Figure 1:**
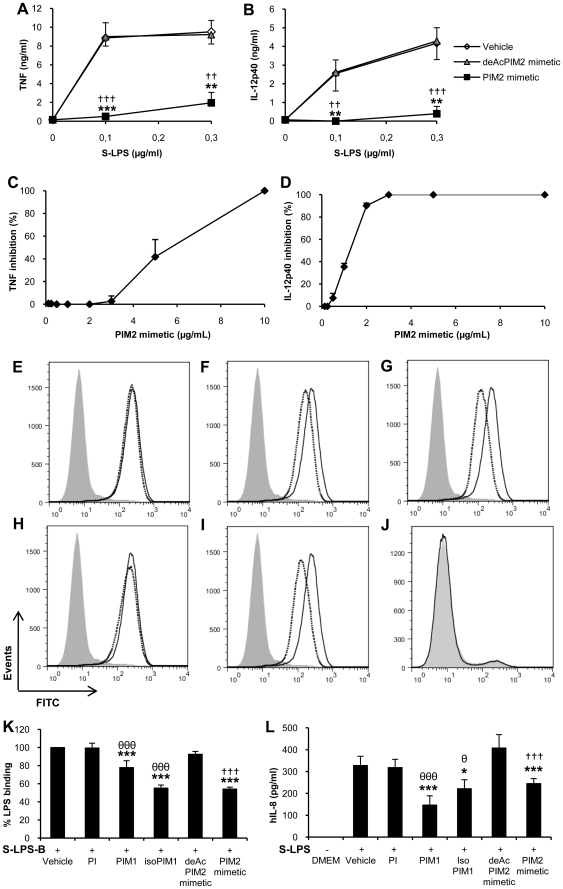
Synthetic PIM analogues inhibit S-LPS-induced responses and binding to HEK cells expressing TLR4, MD2 and CD14. Bone marrow derived macrophages (A, B) were stimulated with increasing concentrations of S-LPS in presence of 10 µg/mL of PIM_2_ mimetic or deAcPIM_2_ mimetic, or vehicle control, and TNF (A) and IL-12 p40 (B) were measured in supernatants after overnight incubation. Results are mean +/− SEM from n = 6 mice from three independent experiments. PIM analogues were titrated (C, D) in the presence of 0.1 µg/mL S-LPS and a 10 µg/mL dose was chosen as this concentration was sufficient for the active PIMs to strongly inhibit LPS-induced TNF (C) and IL-12 p40 (D) release without cytotoxicity. (E–J) HEK cells stably transfected with TLR4, MD2 and CD14 were incubated with PI (E), PIM_1_ (F), isoPIM_1_ (G), deAcPIM_2_ mimetic (H) or PIM_2_ mimetic (I) (10 µg/mL; dotted line) prior to incubation with biotinylated S-LPS (2,5 µg/mL) and streptavidin FITC (black line) or only streptavidin FITC (grey histogram). Non transfected HEK cells were incubated with biotinylated S-LPS as a control (J). Results are from one experiment representative of three independent experiments. (K) Percentage of S-LPS-binding to HEK-MTC cells in presence of vehicle, PI, isoPIM_1_, deAcPIM_2_ mimetic or PIM_2_ mimetic. Results are the mean +/− SD from three independent experiments. (L) Human IL-8 was measured in the supernatant after overnight S-LPS stimulation of HEK-MTC cells. Results are mean +/− SD from triplicates, from one experiment representative of three independent experiments. *, p<0.05; **, p<0.01; ***, p<0.001 versus vehicle; θ, p<0.05; θθθ, p<0.001 indicate significant differences between PIM_1_ or isoPIM_1_ versus PI as control; ††, p<0.01; †††, p<0.001 indicate significant differences between PIM_2_ mimetic and deAcPIM_2_ mimetic as control.

We showed previously that synthetic PIM_1_ and PIM_2_ mimetic (Figure S1) inhibit TNF and IL-12 p40 release by macrophages stimulated with low dose LPS ([19] and Figure 1A, B) at micromolar concentrations (Figure 1C, D). We thus asked whether PIMs could interfere with LPS-binding to cells (Figure 1E–J). By using HEK cells transfected with TLR4, MD2 and CD14, we showed that binding of biotinylated smooth LPS (S-LPS; *E. Coli* serotype O111:B4) was partially inhibited by PIM_1_ (Figure 1F), a PIM_1_ isomer (isoPIM_1_) (Figure 1G) and a PIM_2_ mimetic (Figure 1I) but not by phosphatidyl inositol (PI; Figure 1E) or by a deacylated PIM_2_ mimetic (deAcPIM_2_) control (Figure 1H). Excess of unlabelled S-LPS competed only partially the binding of biotinylated S-LPS (data not shown), although to the same extent as PIMs, indicating a non-saturable, and maybe partially non-specific cellular binding of biotinylated S-LPS. However, no binding of S-LPS-biotin was detected on HEK cells in the absence of TLR4/MD2/CD14 (Figure 1J). The inhibition of S-LPS binding (Figure 1K) was accompanied with an inhibition of IL-8 release by S-LPS-stimulated HEK cells (Figure 1L). PIM_1_, isoPIM_1_ and PIM_2_ mimetic also affected S-LPS-binding to primary macrophages (Figure S2). Thus, there was a partial decrease of S-LPS-biotin binding to TLR4/MD2/CD14 expressing HEK cells as well as primary macrophages in the presence of PIMs.

### Synthetic PIM analogues potently inhibit TLR4 and TLR2 induced pathways

**Figure 2:**
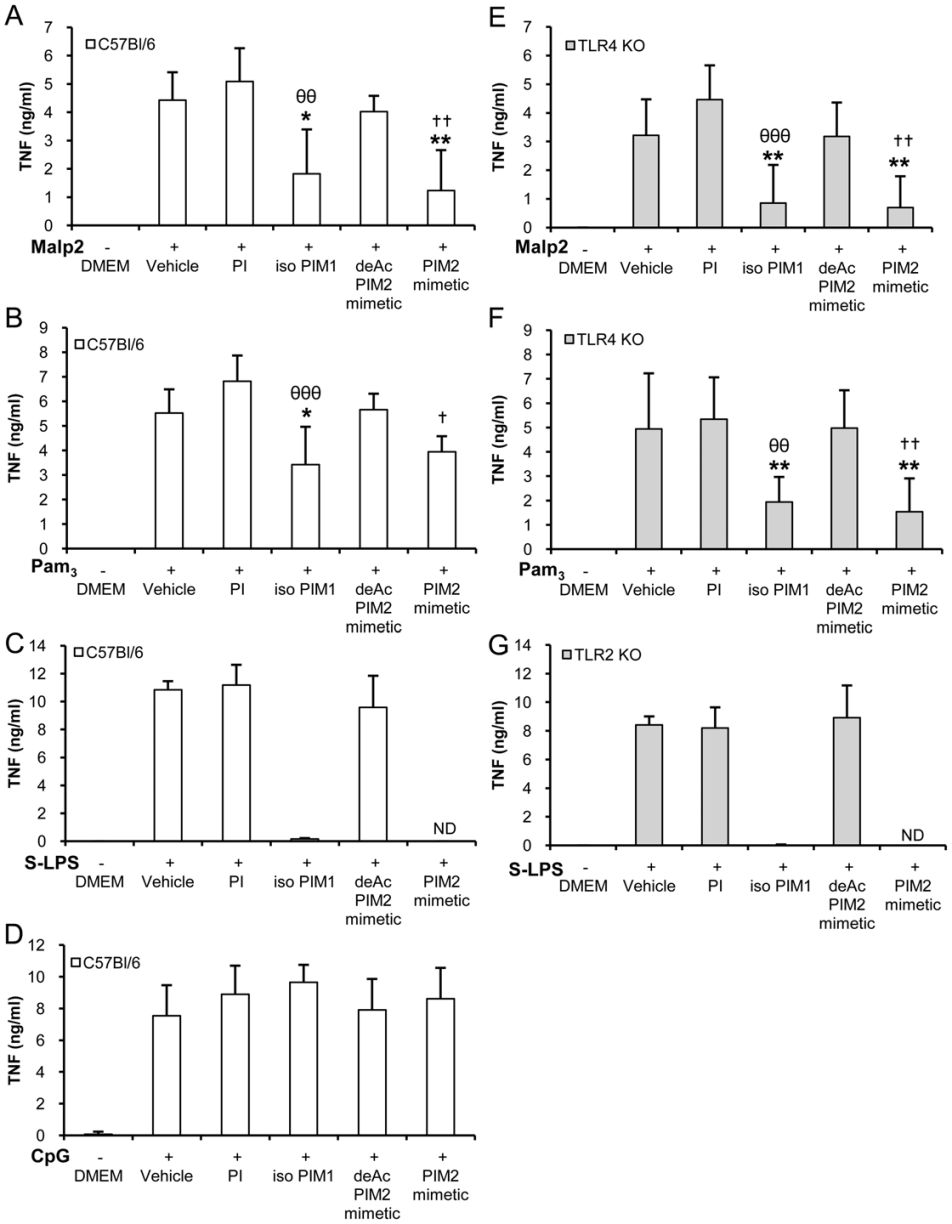
Synthetic PIM analogues inhibit TLR4 and TLR2 pathways but not TLR9. Macrophages from C57Bl/6 (A–D), TLR4 KO (E, F) or TLR2 KO mice (G) were activated with TLR2 agonist Malp2 (A, E) or Pam_3_CSK_4_ (Pam_3_; B, F), TLR4 agonist S-LPS (C, G) or TLR9 agonist CpG (D) in presence of synthetic PI, isoPIM_1_, deAcPIM_2_ mimetic, PIM_2_ mimetic (all at 10 µg/mL), or vehicle. TNF production was measured in the supernatant after overnight incubation. Results are from n = 4–6 mice from two to three independent experiments (A–B, D–F) or n = 2 mice from one experiment representative of two independent experiments (C, G). ND: not detected. *, p<0.05; **, p<0.01 versus vehicle. θθ, p<0.01; θθθ, p<0.001 indicate significant differences between isoPIM_1_ versus PI as control; †, p<0.05; ††, p<0.01 indicate significant differences between PIM_2_ mimetic and deAcPIM_2_ mimetic as control.

We showed previously that the inhibitory effects of the natural PIM_6_ fractions were preferentially targeted to the TLR4 signalling pathway, although the specificity was not absolute for IL-12 p40 release [19]. Using more potent synthetic PIM_1_ and PIM_2_ analogues, we readdressed TLR specificity. Specific TLR4 agonist S-LPS, TLR2/TLR1 agonist Pam_3_CSK_4_, TLR2/TLR6 agonist Malp2, and TLR9 agonist CpG, were used to activate macrophages in the absence or in presence of PIM derivatives. Synthetic isoPIM_1_ and PIM_2_ mimetic inhibited the production of TNF or IL-12 p40 (not shown) after stimulation by Malp2 or Pam_3_CSK_4_ (Figure 2A, B), slightly less potently than they inhibited S-LPS response (Figure 2C), while they did not inhibit TNF release after stimulation by CpG (Figure 2D). Further, the inhibition of Malp2 or Pam_3_CSK_4_ induced TNF could be seen even in the absence of TLR4 (Figure 2E, F) indicating that this effect is independent of the TLR4 pathway. Conversely, the inhibition of S-LPS response could be seen in the absence of TLR2 (Figure 2G). Therefore, the inhibitory effects of synthetic PIM_1_ and PIM_2_ analogues target both TLR2 and TLR4 pathways.

### Not only ampiphilic, acylated TLR agonists, but also Taxol is inhibited by PIM

**Figure 3:**
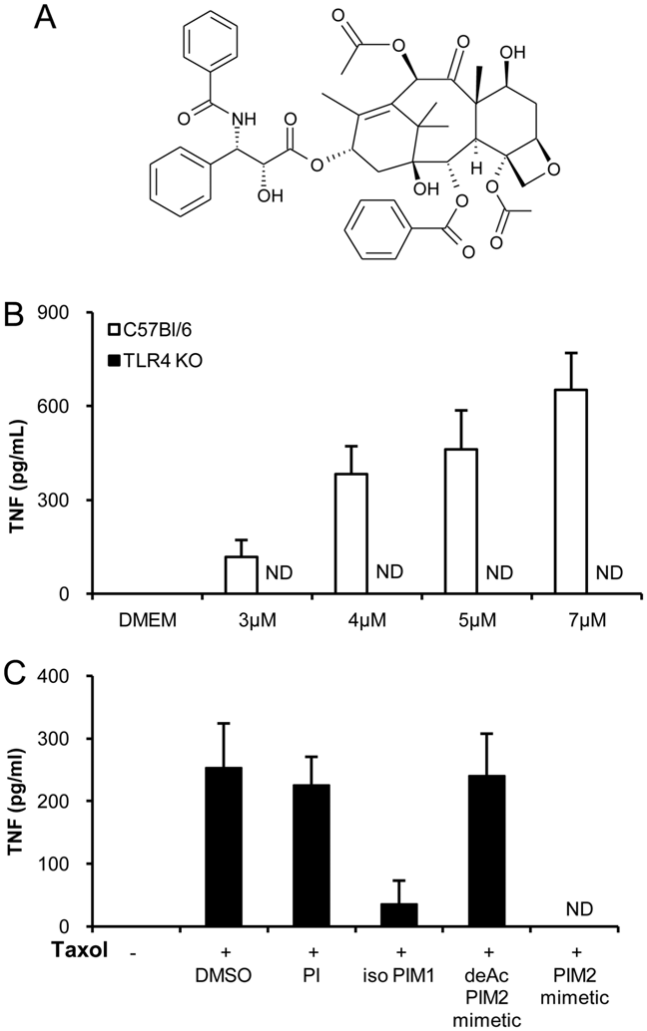
Synthetic PIM analogues inhibit TLR4-dependent, Taxol induced TNF release. The antitumoral compound Taxol (A) was used at increasing concentrations to stimulate macrophages from C57Bl/6 and TLR4 KO mice, showing the TLR4 specificity (B). BMDM from C57Bl/6 mice were stimulated with Taxol (3 µM) in the presence of synthetic PI, isoPIM_1_, deAcPIM_2_ mimetic, PIM_2_ mimetic (all at 10 µg/mL), or vehicle (C) and TNF concentrations were measured in the supernatant after overnight incubation. Results are mean +/− SD from n = 2 mice from one experiment representative of 2 to 3 independent experiments. ND: not detected.

The TLR2 and TLR4 ligands tested above were acylated, amphiphilic molecules. Since LAM were shown to form micelles [47] and PIMs may also do so, we next wanted to exclude that PIMs act by scavenging the different acylated TLR4-agonist S-LPS, or TLR2-agonists Pam_3_CSK_4_ and Malp2. We thus asked whether PIMs could also inhibit macrophage activation triggered by Taxol, a TLR4 agonist of a different molecular class [48], [49]. The contribution of potentially contaminating endotoxins in this stimulation was excluded by pre-incubating Taxol with polymyxin B at a concentration sufficient to neutralise 100 ng/mL of LPS (data not shown). As shown in Figure 3A, Taxol is not acylated, it requires the presence of TLR4 to stimulate TNF release by bone marrow-derived macrophages (Figure 3B), and Taxol stimulation is potently inhibited by isoPIM_1_ and PIM_2_ mimetic but not by PI and deAcPIM_2_ mimetic controls (Figure 3C). Thus, a TLR4 ligand unlikely to form micelles is also susceptible to PIM inhibition.

### Interference of PIM analogues with smooth LPS binding to CD14

Since synthetic PIM analogues could target both TLR2 and TLR4 pathways we hypothesized that they may interact with a co-receptor common to TLR2 and TLR4. CD14 was a likely candidate. Indeed, natural PIM_2_ from *M. Kansasii* was shown to interact with CD14 [50] and S-LPS binding was shown to depend on the presence of CD14 [51]. We first confirmed that S-LPS binding depended on the presence of CD14 as S-LPS binding was essentially absent in CD14-deficient BMDM macrophages, while it was only slightly reduced in TLR4-deficient macrophages and similar in MD2-deficient and wild-type macrophages (Figure S3).

**Figure 4:**
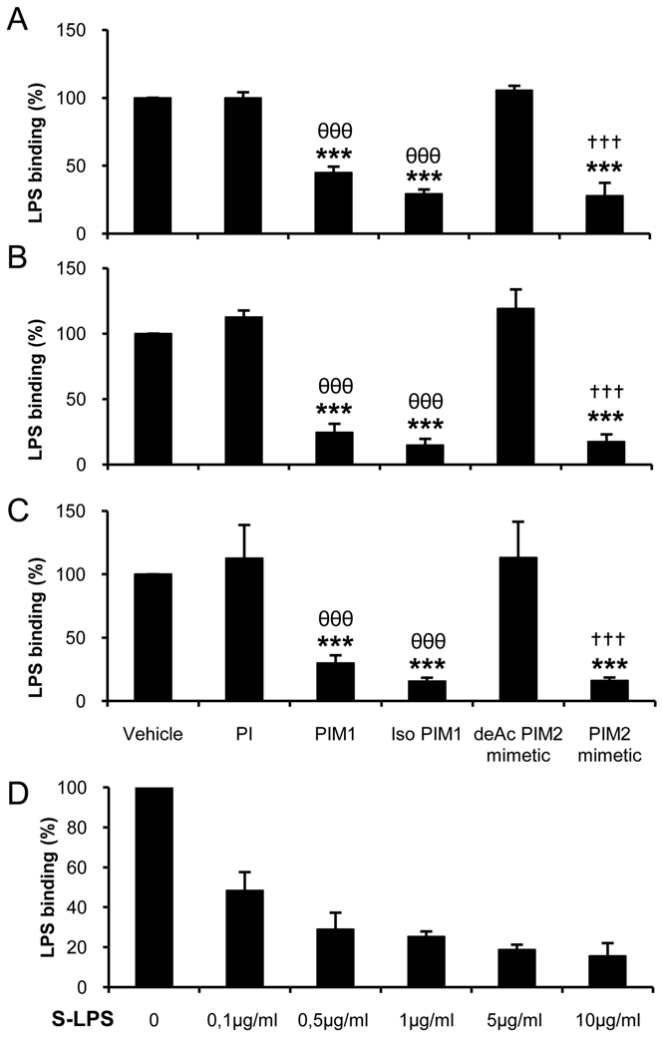
Synthetic PIM analogues inhibit S-LPS-binding to soluble CD14. The effects of PIMs on the binding of biotinylated S-LPS to sCD14 was investigated in presence of 1% FCS (A, D), or 0.1% serum from wild-type mice (B) or from LBP-deficient mice (C). Solid phase adsorbed sCD14 was incubated (1 hr at 37°C) in the presence of synthetic PI, PIM_1_, isoPIM_1_, deAcPIM_2_ mimetic and PIM_2_ mimetic (all at 10 µg/mL) or vehicle, before addition of biotinylated S-LPS (0.1 µg/mL; 2 hrs at 37°C). Binding specificity was determined by incubation with increasing concentrations of non biotinylated S-LPS 1 hr prior to biotinylated S-LPS (D). Results are expressed as percentage of biotinylated S-LPS-binding to sCD14 as compared to incubation with vehicle and are mean +/− SD from three independent experiments. ***, p<0.001 versus vehicle; θθθ, p<0.001 indicate significant differences between isoPIM_1_ versus PI as control; †††, p<0.001 indicate significant differences between PIM_2_ mimetic and deAcPIM_2_ mimetic as control.

We then tested directly the ability of PIMs to interfere with S-LPS binding to soluble CD14 (sCD14) in presence of serum. Indeed, LPS-binding protein (LBP) present in serum increases LPS binding to sCD14 (not shown; [52]). S-LPS-biotin binding to sCD14 coated on a solid phase was strongly inhibited by PIM_1_, isoPIM_1_ and PIM_2_ mimetic but not by PI and deAcPIM_2_ mimetic controls (Figure 4). To avoid the contribution of LBP in this interaction, we compared S-LPS-binding to sCD14 in fetal calf serum (Figure 4A), or in serum from wild-type (Figure 4B) or LBP-deficient mice (Figure 4C). Inhibition of S-LPS-biotin binding to sCD14 by PIM_1_, isoPIM_1_ and PIM_2_ mimetic but not by PI and deAcPIM_2_ mimetic controls occurred in mouse serum from wild-type or LBP-deficient mice, thus in the presence or in the absence of LBP. The inhibition of S-LPS binding to sCD14 by PIM_1_ was slightly weaker than the inhibition by isoPIM_1_ or PIM_2_ mimetic, similar to the effect seen on whole cell S-LPS-binding. Binding of biotinylated S-LPS was effectively competed by unlabelled S-LPS in this system (Figure 4D).

To address the functional relevance of this interaction, we asked whether soluble CD14 might “scavenge” some PIM molecules and reduce PIM inhibition on S-LPS-induced TNF response. Addition of sCD14 had essentially no effect on isoPIM_1_ or PIM_2_ mimetic inhibition of S-LPS-induced TNF (Figure S4A). Soluble CD14 was used at a concentration effective for restoring some S-LPS functional effect in CD14-deficient macrophages (Figure S4B). Therefore, anti-inflammatory PIMs can prevent S-LPS binding to sCD14, in an LBP-independent way, but CD14 may not be directly involved in PIM inhibitory effects.

### PIM inhibition of TLR2-induced cytokine responses is independent of CD14

**Figure 5:**
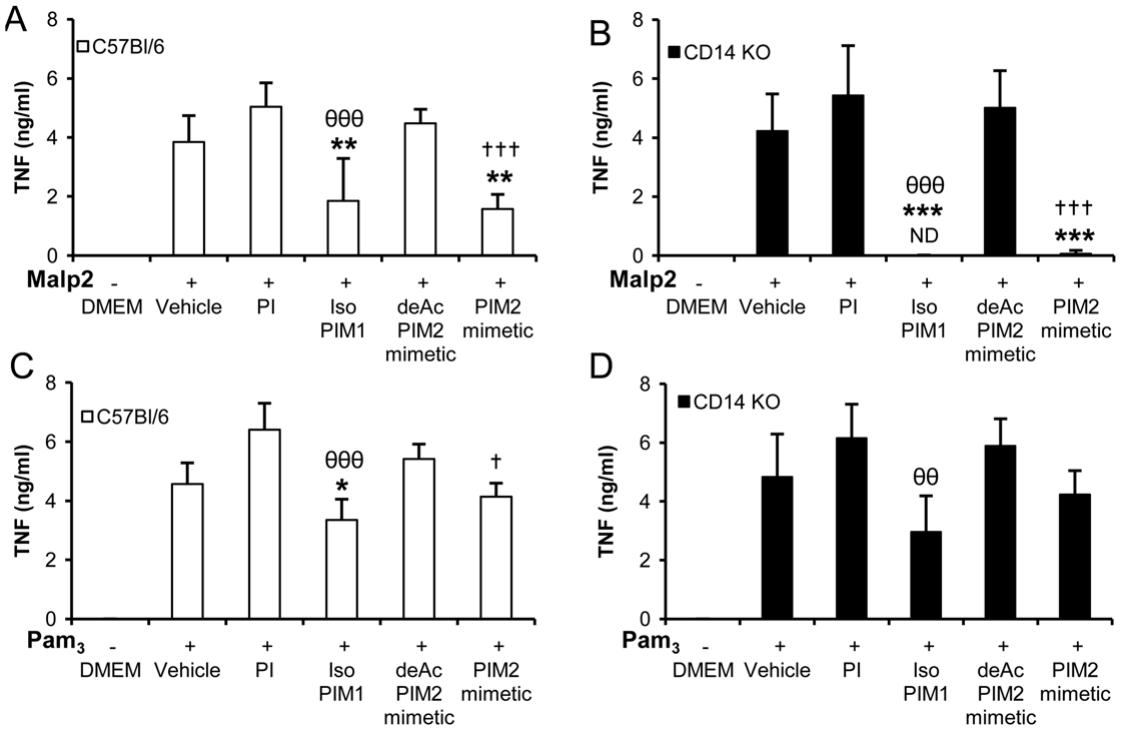
Inhibition of TLR2 signaling by PIM analogues is independent of CD14. Macrophages from C57Bl/6 mice (A, C) or CD14 KO mice (B, D) were incubated with synthetic PI, isoPIM_1_, deAcPIM_2_ mimetic, PIM_2_ mimetic (10 µg/mL) or control vehicle prior to stimulation with Malp2 (30 ng/mL; A, B) or Pam_3_CSK_4_ (Pam_3_; 0.5 µg/mL; C, D). TNF release was measured in supernatants after overnight incubation. Results are mean +/− SD from n = 4 mice from two independent experiments. *, p<0.05; **, p<0.01; ***, p<0.001 versus vehicle. θθ, p<0.01; θθθ, p<0.001 indicate significant differences between isoPIM_1_ versus PI as control; †, p<0.05; †††, p<0.001, indicate significant differences between PIM_2_ mimetic versus deAcPIM_2_ mimetic as control.

CD14 is able to recognize different ligands beside LPS and it has been involved in TLR2-signaling induction in response to zymosan or *Listeria monocytogenes* [42], [53]. Therefore, we next asked whether CD14 was an obligatory co-receptor for the anti-inflammatory effects of PIMs on the TLR2 pathways. By using macrophages from wild-type or CD14 KO mice, we show that TNF stimulation by TLR2/TLR6 agonist Malp2 and TLR2/TLR1 agonist Pam_3_CSK_4_ is retained in the absence of CD14 (Figure 5A–D). PIM analogues isoPIM_1_ and PIM_2_ mimetic, but neither PI nor deAcPIM_2_ mimetic controls, inhibit these TLR2-induced TNF responses both in wild type (Figure 5A, C) and in CD14-deficient macrophages (Figure 5B, D). Similar results were obtained for inhibition of IL-12 p40 release (data not shown). Therefore, the inhibitory effects of synthetic PIM_1_ and PIM_2_ analogues on the TLR2 pathways are independent of CD14.

### CD14 requirement is associated with PIM inhibition of TNF, while CD14 is dispensable for PIM inhibition of IL-12 p40 release, after LPS/TLR4 activation

**Figure 6:**
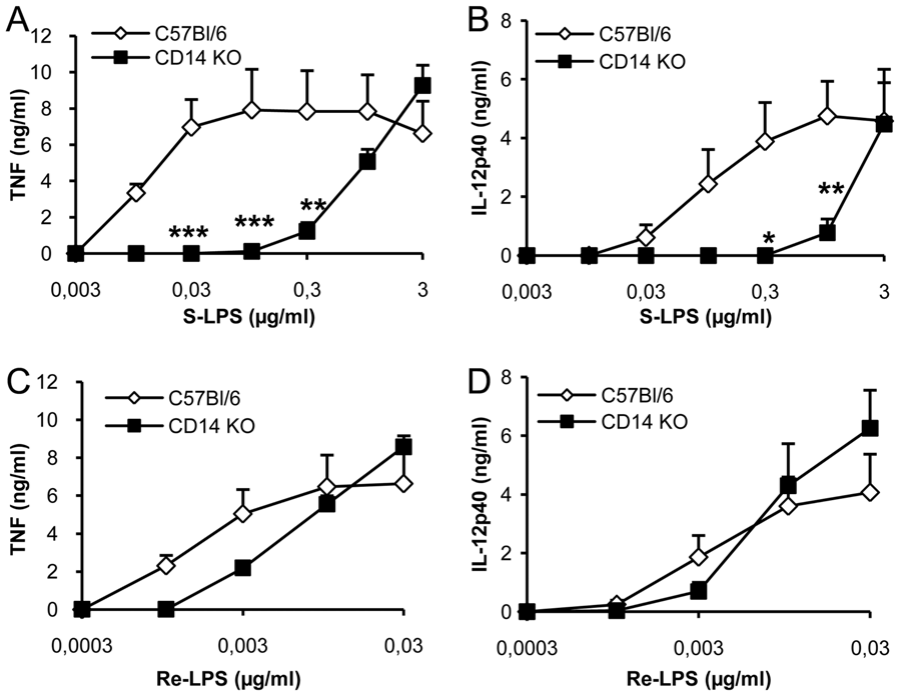
CD14-dependency of TNF and IL-12 p40 release induced by S-LPS versus Re-LPS. Macrophages from C57Bl/6 or CD14 KO mice were stimulated with increasing concentrations of S-LPS (A, B) or Re-LPS (C, D). TNF (A, C) and IL-12 p40 (B, D) concentrations were measured in the supernatants after overnight incubation. Results are mean +/− SEM from n = 4 mice from two independent experiments. *, p<0.05; **, p<0.01; ***, p<0.001 indicate significant differences between C57Bl/6 and CD14 KO.

Concerning TLR4 pathway, CD14 has been shown to be essential for the cellular binding and activity of smooth, S-LPS, while rough LPS (Re-LPS) may bind and activate cells independently of the presence of CD14 [41], [42]. Using macrophages derived from CD14-deficient mice, we confirmed that S-LPS induced a CD14-dependent release of TNF and IL-12 p40 at low concentrations while concentrations of S-LPS above 1 µg/mL induced TNF and IL-12 p40 release in the absence of CD14 (Figure 6A, B; [54], [55]). Consistent with previous reports, Re-LPS uses a CD14-independent pathway to induce TNF and IL-12 p40 (Figure 6C, D).

**Figure 7:**
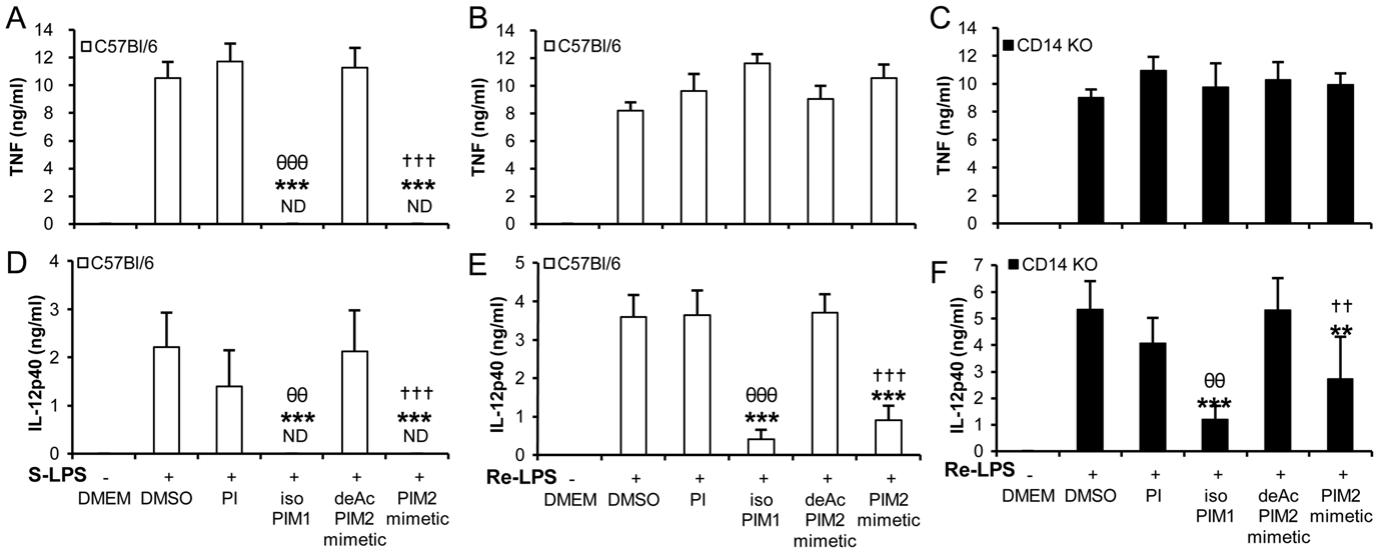
Differential inhibition of S-LPS versus Re-LPS induced TNF and IL-12 p40 release by PIMs. Concentrations of TNF (A–C) and IL-12 p40 (D–F) in supernatants of wild type (A, B, D, E) or CD14-deficient (C, F) macrophages stimulated overnight with 100 ng/mL of S-LPS (A, D) or Re-LPS (B, C, E, F) in the presence of synthetic PI, isoPIM_1_, deAcPIM_2_ mimetic, PIM_2_ mimetic (10 µg/mL), or vehicle. Results are mean +/− SD from n = 4 mice from two independent experiments representative of three independent experiments. ND: not detected. **, p<0.01; ***, p<0.001 versus vehicle. θθ, p<0.01; θθθ, p<0.001 indicate significant differences between isoPIM_1_ versus PI as control; ††, p<0.01; †††, p<0.001 indicate significant differences between PIM_2_ mimetic versus deAcPIM_2_ mimetic as control.

We then analyzed the role of CD14 in PIM inhibition of TLR4 pathway by comparing S- and Re-LPS responses. While S-LPS-induced TNF production was strongly inhibited by isoPIM_1_ and PIM_2_ mimetic (Figure 7A), CD14-independent TNF production induced after Re-LPS stimulation was not inhibited by isoPIM_1_ and PIM_2_ mimetic, neither in wild type (Figure 7B) nor in CD14-deficient macrophages (Figure 7C). In contrast, IL-12 p40 production induced either in a CD14-dependent manner by S-LPS (Figure 7D), or in a CD14-independent manner with Re-LPS (Figure 7E), was potently inhibited by isoPIM_1_ and PIM_2_ mimetic. PIM anti-inflammatory effect on IL-12 p40 was not dependent on the presence of CD14, since CD14 independent activation by TLR4 agonist Re-LPS was also reduced by isoPIM_1_ and PIM_2_ mimetic in CD14 deficient macrophages (Figure 7F). Interestingly, both CD14-dependent S-LPS induced TNF and IL-12 p40 were inhibited by active PIMs, while CD14-independent Re-LPS-induced IL-12 p40, but not TNF release, was inhibited by isoPIM_1_ and PIM_2_ mimetic. This was not merely a titration effect, since even at lower doses of 3 to 10 ng/mL of Re-LPS were isoPIM_1_ and PIM_2_ mimetic inhibiting IL-12 p40 release while TNF response was spared (Figure S5). The data indicated multifold effects of PIMs on these pathways.

**Figure 8:**
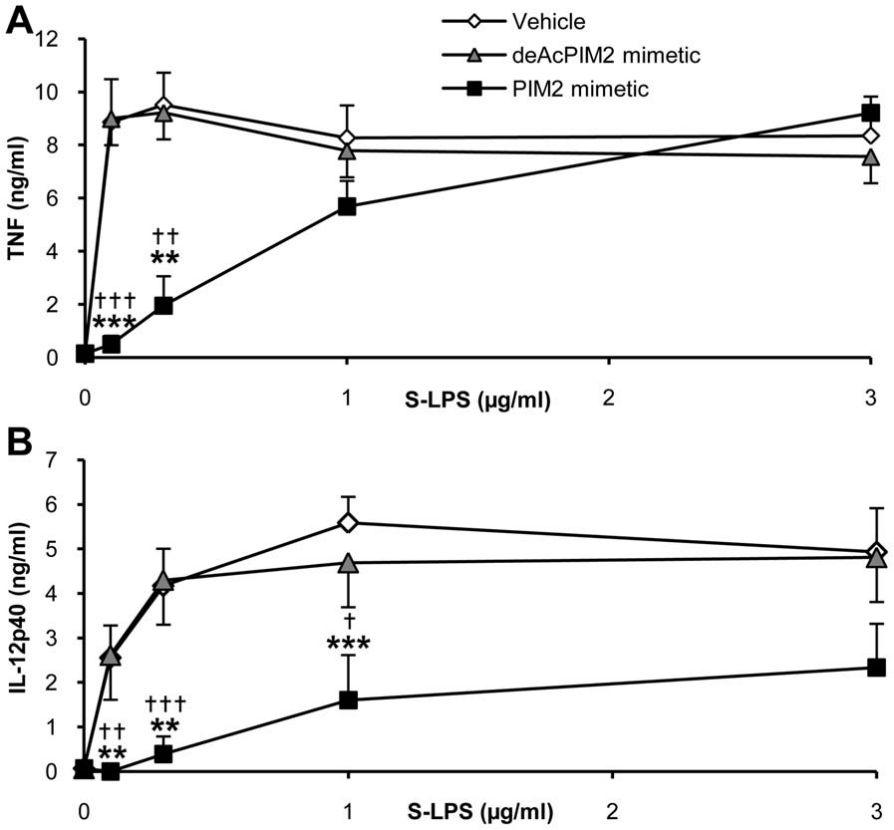
High S-LPS concentrations prevent TNF, but not IL-12 p40 inhibition by PIM_2_ analogue. Macrophages from C57Bl/6 mice were stimulated with increasing doses of S-LPS in the presence of 10 µg/mL of deAcPIM_2_ mimetic and PIM_2_ mimetic or vehicle control. After overnight incubation, TNF (A) and IL-12 p40 (B) concentrations were measured in supernatants. Results are mean +/− SEM from n = 6 mice from three independent experiments. ***, p<0.001 versus vehicle. †, p<0.05 indicate significant differences between PIM_2_ mimetic and deAcPIM_2_ mimetic.

To further address the role of CD14 in the PIM inhibition of S-LPS-induced response, we next investigated PIM anti-inflammatory effect on the CD14-independent stimulation by high S-LPS concentrations. Interestingly, TNF release stimulated by 1–3 ug/mL of S-LPS, in a CD14-independent way (see Figure 6), was not inhibited by PIM_2_ mimetic (Figure 8A), while CD14 independent release of IL-12 p40 induced by S-LPS at 1–3 ug/mL concentrations was strongly inhibited by PIM_2_ mimetic (Figure 8B). When titrated in parallel by increasing the ratio of S-LPS over PIM concentrations, TNF release was clearly less inhibited than IL-12 p40 release.

Thus, the fact that both CD14-independent Re-LPS-induced IL-12 p40 release and CD14-independent, high dose S-LPS-induced IL-12 p40 were inhibited by isoPIM_1_ or PIM_2_ mimetic indicated that PIMs affect IL-12 p40 release independently of CD14. Conversely, the fact that both CD14-independent Re-LPS-induced TNF release and CD14-independent, high dose S-LPS-induced TNF were not inhibited by isoPIM_1_ and PIM_2_ mimetic, while CD14-dependent S-LPS induced TNF was inhibited, suggested that PIM inhibition of TNF release targeted a CD14-dependent pathway.

## Discussion

Bacterial pathogens have developed numerous strategies to undermine host innate responses and promote infection [56], [57]. PRRs such as TLR2 or TLR4 are crucial to detect different PAMPs and to coordinate signals that allow host cells to induce a range of defence mechanisms, including oxidative stress, autophagy and cell death. However, PRRs are also targets for microorganisms to subvert both immune recognition and intracellular signalling. Here we show that PIM1 and PIM2 analogues interfere with the pathways activated by both TLR2 and TLR4. *M. tuberculosis* and *M. bovis* were also shown to trigger TLR2 and TLR4 pathways and produce TLR2 but also TLR4 agonists such as *M. bovis* tetra-acylated LM or *M. tuberculosis* LM [19]. Mycobacteria thus produce on the one hand PAMPs that are recognized by the host, and on the other hand molecules that can interfere with the host innate immune responses, with a possible balance between those. Indeed, PIMs inhibit macrophage activation by *M. tuberculosis* LM [19].

We reported previously that some natural and synthetic PIMs inhibit the expression of NO, a potent mycobactericidal mediator, and of pro-inflammatory cytokines essential for host response to mycobacteria such as TNF, IL-12p40 and IL-1 in vitro and in vivo in response to LPS [19]. In line with this, natural or synthetic PIMs [33], [34] or a synthetic PIM_2_ analogue [35] suppress ovalbumin-induced allergic airway eosinophilia, a model in which LPS contaminant has been shown to play a crucial role [32]. Here, in order to further understand the role that PIMs may play in immune evasion, we thus addressed molecular mechanisms involved in the inhibition by PIM analogues of LPS pro-inflammatory responses.

PIMs were described as mycobacterial adhesins mediating binding to mammalian cells, but this effect was mostly attributed to high order, polar PIM_5_ or PIM_6_ [58]. PIMs interact with several cell surface receptors, including not only TLR2 but also CD1d [59], [60], and C-type lectins mannose receptor or DC-SIGN [31]. However, we showed previously that synthetic PIM_1_ or PIM_2_ mimetic analogues are not TLR2 agonists as they do not trigger inflammatory responses at micromolar concentrations, and that CD1d, mannose receptor and SIGN-R1 are dispensable for PIM inhibition of LPS-induced pro-inflammatory response in murine macrophages [19]. We thus asked whether ‘anti-inflammatory’ PIM analogues could compete with LPS for binding on target cells. Using flow cytometry to quantify LPS-binding to HEK cells expressing LPS receptor and co-receptors TLR4, MD2 and CD14 or to primary macrophages, we show that anti-inflammatory PIM_1_, isoPIM_1_ and PIM_2_ mimetic partially inhibited the binding of biotinylated S-LPS to cells while inactive controls PI and non-acylated, deAcPIM_2_ mimetic did not. The extent of competition achieved with active PIM_1_ and PIM_2_ analogues was similar to that observed with an excess of unlabelled S-LPS, although incomplete, which might indicate some non-saturable and potentially non-specific cellular binding of biotinylated S-LPS. Increased internalization of TLR4 was unlikely responsible for the decreased S-LPS-binding by PIMs. Indeed, PIMs prevented the down-regulation of TLR4 mRNA expression seen 2 h after S-LPS-stimulation (data not shown). Furthermore, macrophage pre-treatment with cytochalasin D did not affect PIMs inhibitory activities (data not shown).

Natural PIMs inhibited preferentially the TLR4 pathway [19], suggesting a specific interaction of the PIMs with TLR4 or TLR4 pathway. However, using more active, synthetic PIM analogues we demonstrated PIM inhibitory effects on macrophage responses to either TLR2 or TLR4 agonists. The inhibition of TLR2/TLR1 agonist Pam_3_CSK_4_ and TLR2/TLR6 agonist Malp2 induced responses occurred even in the absence of TLR4 and, conversely, the inhibition of TLR4 agonist S-LPS response occurred in the absence of TLR2. These results indicated that cytokine responses to both TLR2 and 4 pathways can be inhibited by active PIMs and suggested that PIMs were unlikely to act through an exclusive interaction with TLR4. We hypothesised that PIMs may target a co-receptor common to both TLR2 and TLR4. Since PIMs are GPI-anchor, ampiphilic structures with acylated moieties, they might interfere with the organization of supramolecular coreceptors/receptors multimeric complexes involved in both TLR2 and TLR4 pathways. Indeed, LAM GPI anchor PIM_6_ competitively inhibit the insertion of LAM into mononuclear cell plasma membranes, likely into specialized domains enriched in endogenous GPI-anchored molecules [36]. LAM were shown to modify the signalling machineries of rafts/microdomains [37]. We investigated CD14, one of the GPI-anchored proteins present in hematopoietic cell microdomains, as a potential target candidate for PIMs effect on the TLR2 and TLR4 pathways. Indeed, CD14 is necessary for S-LPS binding to cells and subsequent signalling [51] and CD14 was also implicated as a first step in Pam_3_CSK_4_ recognition, inducing physical proximity with TLR2/TLR1 and formation of the TLR2 signalling complex [61]. Natural PIM_2_ from *M. kansasii* was shown to interact with CD14 [50], and CD14 was implicated in mycobacterial LM and H37Ra LAM pro-inflammatory activities [54], [62]. Here, we documented the inhibition of S-LPS binding to soluble CD14 by the anti-inflammatory PIM_1_, isoPIM_1_ and PIM_2_ mimetic, but not by PI or a deacylated PIM_2_ analogue. Thus, PIM derivatives interfered with S-LPS binding to cells, and S-LPS-interaction with CD14 was a likely target for this inhibition. However, PIM inhibition of S-LPS-interaction with sCD14 was independent of the presence of LBP. Further, PIM inhibition of S-LPS-induced TNF release was not restored by addition of soluble CD14 to cells, indicating that PIMs might not directly compete with S-LPS for binding to CD14, but might rather affect an earlier step independent of LBP. Indeed, several receptors found in serum are involved in LPS disaggregation like HMGB1 [63], and might be considered.

We then addressed the functional implication of CD14 in PIM anti-inflammatory effects by using macrophages deficient for CD14. A partial CD14-dependency was reported for Malp2, but not for Pam_3_CSK_4_, induced TNF response [42], while in our hands Malp2-induced TNF release was CD14 independent. The CD14 independent activation of TLR2 agonists Malp2 and Pam_3_CSK_4_ was reduced by isoPIM_1_ and PIM_2_ mimetic, indicating that active PIMs inhibit TLR2 signalling pathways by a mechanism independent of CD14. We next asked whether PIM interference with LPS-CD14 was a necessary component of the functional inhibition of LPS-induced pro-inflammatory responses by PIMs, at different levels. Active PIM analogues inhibited CD14-independent Re-LPS-induced IL-12 p40 as well as CD14-independent IL-12 p40 stimulation induced by high S-LPS concentrations. However, while CD14-dependent TNF release was potently inhibited by PIM_1_ and PIM_2_ analogues, neither CD14-independent Re-LPS induced TNF release, nor CD14-independent, high dose S-LPS-induced TNF were affected by PIMs. Thus, CD14-independent IL-12 p40 release was inhibited by PIM_1_ and PIM_2_ derivatives, while the CD14-independent TNF release was not. These data suggest that PIMs affected IL-12 p40 release independently of CD14 while PIMs targeted a CD14-dependent pathway for inhibition of TNF release.

We propose that PIMs may exert their inhibitory activity through different ways, by inhibiting S-LPS binding to CD14, and by interfering at another level. Indeed, CD14 participates in LPS-induced TNF production in RAW cells and peritoneal macrophages while a CD14-independent pathway is used in Kupffer cells [64]. Further, although CD14 is essential for cell binding and activity of low dose smooth LPS, CD14 is dispensable at high doses of S-LPS and for binding and cell activation by rough LPS [41], [42], confirming that TLR4 ligands can induce TNF and IL-12 production by different mechanisms which might not be equally affected by PIMs. IL-12 p40 release after S-LPS stimulation requires CD14 in macrophages, but other receptors such as CD11b and CD18 (Mac-1) have been involved in the optimal expression of IL-12 p40 and IL-12 p35 genes in response to LPS or Taxol [65]. The regulation of IL-12 p40 expression is complex [66]. One major regulator of IL-12 p40 production is the anti-inflammatory cytokine IL-10. We showed previously that PIM inhibitory activity was not dependent on an increase in IL-10 expression as this cytokine is also inhibited by PIMs [19]. Combined activation of TLRs and other pattern recognition receptors or co-receptors may result in agonistic or antagonistic interactions and, in particular, the regulation of IL-12 expression in response to TLR trigger is the net result of complex activation and down-regulations implicating different kinases such as PI3K or AKT (reviewed in [66], [67]). The potential interference of PIMs with other mechanisms or signalling pathways involved in the expression of IL-12 will require further investigations.

In conclusion, as summarized schematically in Figure S6, we show that PIMs inhibit macrophage activation in response to TLR2 or TLR4 pathways at different levels. PIMs block LPS binding to CD14, which may explain PIM inhibition of CD14-dependent LPS functional responses through TLR4. However, not all TLR responses need CD14, and this is particularly so for TLR4 response to rough LPS or to high dose smooth LPS, but also for some TLR2 responses. In these cases, PIM inhibitory effect has to be explained at another level, likely downstream of TLRs.

## Supporting Information

Figure S1 **Structure of synthetic PIM_1_, isoPIM_1_ and PIM_2_ mimetics.** Schematic representation of synthetic PIM_1_ showing the C16 and C18 acyl groups on glycerol chain positions sn-2 and sn-1, an isomer of PIM_1_ (isoPIM_1_) carrying the phosphatidyl group at position O-2 and the mannosyl residue at O-1 of D-*myo*-inositol, the precursor PI, a synthetic mimetic of PIM_2_ (PIM_2_ mimetic) bearing C16 and C18 acyl chains, and the de-acylated precursor of the PIM_2_ mimetic (deAcPIM_2_ mimetic) as control molecule.
(TIF)

Figure S2 **Synthetic PIM analogues inhibit S-LPS-binding to macrophages.** Bone marrow derived macrophages from C57Bl/6 mice were incubated with 10 µg/mL (dotted line) deAcPIM_2_ mimetic (A) or PIM_2_ mimetic (B) prior incubation with 5 µg/mL of biotinylated S-LPS and streptavidine FITC (black line). DeAcPIM_2_ mimetic did not displace S-LPS and was superimposed with S-LPS plus vehicle (A). In controls, macrophages were stained only with streptavidin FITC (grey histogram). Results shown are from cells derived from one mouse representative of cells from four different mice. (C) Percentage of S-LPS binding to macrophages in presence of PIMs or vehicle. Mean +/− SD from n = 4 mice from 2 independent experiments. ***, p<0.001 versus vehicle. †††, p<0.001 indicate significant differences between deAcPIM_2_ mimetic and PIM_2_ mimetic.
(TIF)

Figure S3 **CD14 is an important co-receptor for S-LPS-binding to macrophages.** Bone marrow derived macrophages from C57Bl/6 (A), TLR4 KO (B), MD2 KO (C) or CD14 KO (D) mice were incubated with biotinylated S-LPS and streptavidine FITC (black line). In controls, macrophages were only incubated with streptavidine FITC (grey histogram). Results are from one mouse representative of four mice. (E) Percentage of S-LPS-binding to macrophages compared to C57Bl/6 binding level. Mean +/− SD from n = 4–8 mice from two to four independent experiments. **, p<0.01, ***, p<0.001 versus C57Bl/6.
(TIF)

Figure S4 **Addition of sCD14 does not affect PIM inhibition of S-LPS-induced TNF.** (A) Macrophages from C57Bl/6 mice were incubated with murine soluble CD14 (sCD14; 5 µg/mL) and PIMs (10 µg/mL) as indicated prior to stimulation with S-LPS (100 ng/mL). (B) Wild type or CD14 KO macrophages were stimulated with S-LPS in the absence or in the presence of murine soluble CD14 (sCD14; 5 µg/mL). TNF concentration was measured in the supernatants after overnight incubation. Mean +/− SD from n = 4 mice from two experiments representative of three independent experiments. ***, p<0.001 versus vehicle. θθθ, p<0.001 indicate significant differences between isoPIM_1_ versus PI as control, †††, p<0.001 indicate significant differences between deAcPIM_2_ mimetic and PIM_2_ mimetic.
(TIF)

Figure S5 **Differential inhibition of induced TNF and IL-12 p40 release by PIMs at low doses of Re-LPS.** Concentrations of TNF (A) and IL-12 p40 (B) in supernatants of CD14-deficient macrophages stimulated overnight with 3 or 10 ng/mL of Re-LPS in the presence of synthetic isoPIM_1_, deAcPIM_2_ mimetic, PIM_2_ mimetic (10 µg/mL), or vehicle. Results are mean +/− SD from n = 2 mice.
(TIF)

Figure S6 **Schematic model of PIM interference with TLR2 and TLR4 responses.** PIMs block LPS binding to CD14, which may explain the inhibition of PIM in CD14-dependent LPS functional responses through TLR4. However, not all TLR responses need CD14, as indicated for TLR4 response to rough LPS or to high micromolar doses of smooth LPS, but also for TLR2/TLR1 response to Pam_3_CSK_4_ and TLR2/TLR6 response to Malp2. In these cases, PIM inhibitory effect may be downstream of TLRs. In addition, IL-12p40 expression requires other surface molecules to be complete, such as CD11b and CD18, and this may in part explain the different sensitivity of TNF and IL-12p40 to the inhibition by PIMs.
(TIF)

## References

[1] Flynn JL, Goldstein MM, Chan J, Triebold KJ, Pfeffer K (1995). Tumor necrosis factor-alpha is required in the protective immune response against Mycobacterium tuberculosis in mice.. Immunity.

[2] Bean AG, Roach DR, Briscoe H, France MP, Korner H (1999). Structural deficiencies in granuloma formation in TNF gene-targeted mice underlie the heightened susceptibility to aerosol Mycobacterium tuberculosis infection, which is not compensated for by lymphotoxin.. J Immunol.

[3] Flynn JL, Chan J (2001). Immunology of tuberculosis.. Annu Rev Immunol.

[4] Flynn JL (2006). Lessons from experimental Mycobacterium tuberculosis infections.. Microbes Infect.

[5] Cooper AM (2009). Cell-mediated immune responses in tuberculosis.. Annu Rev Immunol.

[6] Cooper AM, Kipnis A, Turner J, Magram J, Ferrante J (2002). Mice lacking bioactive IL-12 can generate protective, antigen-specific cellular responses to mycobacterial infection only if the IL-12 p40 subunit is present.. J Immunol.

[7] Altare F, Durandy A, Lammas D, Emile JF, Lamhamedi S (1998). Impairment of mycobacterial immunity in human interleukin-12 receptor deficiency.. Science.

[8] Jouanguy E, Lamhamedi-Cherradi S, Altare F, Fondaneche MC, Tuerlinckx D (1997). Partial interferon-gamma receptor 1 deficiency in a child with tuberculoid bacillus Calmette-Guerin infection and a sibling with clinical tuberculosis.. J Clin Invest.

[9] Fremond CM, Togbe D, Doz E, Rose S, Vasseur V (2007). IL-1 receptor-mediated signal is an essential component of MyD88-dependent innate response to Mycobacterium tuberculosis infection.. J Immunol.

[10] Knutson KL, Hmama Z, Herrera-Velit P, Rochford R, Reiner NE (1998). Lipoarabinomannan of Mycobacterium tuberculosis promotes protein tyrosine dephosphorylation and inhibition of mitogen-activated protein kinase in human mononuclear phagocytes. Role of the Src homology 2 containing tyrosine phosphatase 1.. J Biol Chem.

[11] Tailleux L, Pham-Thi N, Bergeron-Lafaurie A, Herrmann JL, Charles P (2005). DC-SIGN induction in alveolar macrophages defines privileged target host cells for mycobacteria in patients with tuberculosis.. PLoS Med.

[12] Pathak SK, Basu S, Bhattacharyya A, Pathak S, Kundu M (2005). Mycobacterium tuberculosis lipoarabinomannan-mediated IRAK-M induction negatively regulates Toll-like receptor-dependent interleukin-12 p40 production in macrophages.. J Biol Chem.

[13] Nigou J, Zelle-Rieser C, Gilleron M, Thurnher M, Puzo G (2001). Mannosylated lipoarabinomannans inhibit IL-12 production by human dendritic cells: evidence for a negative signal delivered through the mannose receptor.. J Immunol.

[14] Johansson U, Ivanyi J, Londei M (2001). Inhibition of IL-12 production in human dendritic cells matured in the presence of Bacillus Calmette-Guerin or lipoarabinomannan.. Immunol Lett.

[15] Geijtenbeek TB, Van Vliet SJ, Koppel EA, Sanchez-Hernandez M, Vandenbroucke-Grauls CM (2003). Mycobacteria target DC-SIGN to suppress dendritic cell function.. J Exp Med.

[16] Gringhuis SI, den Dunnen J, Litjens M, van Het Hof B, van Kooyk Y (2007). C-type lectin DC-SIGN modulates Toll-like receptor signaling via Raf-1 kinase-dependent acetylation of transcription factor NF-kappaB.. Immunity.

[17] Quesniaux V, Fremond C, Jacobs M, Parida S, Nicolle D (2004). Toll-like receptor pathways in the immune responses to mycobacteria.. Microbes Infect.

[18] Doz E, Rose S, Nigou J, Gilleron M, Puzo G (2007). Acylation determines the toll-like receptor (TLR)-dependent positive versus TLR2-, mannose receptor-, and SIGNR1-independent negative regulation of pro-inflammatory cytokines by mycobacterial lipomannan.. J Biol Chem.

[19] Doz E, Rose S, Court N, Front S, Vasseur V (2009). Mycobacterial phosphatidylinositol mannosides negatively regulate host Toll-like receptor 4, MyD88-dependent proinflammatory cytokines, and TRIF-dependent co-stimulatory molecule expression.. J Biol Chem.

[20] Schlesinger LS, Hull SR, Kaufman TM (1994). Binding of the terminal mannosyl units of lipoarabinomannan from a virulent strain of Mycobacterium tuberculosis to human macrophages.. J Immunol.

[21] Kang BK, Schlesinger LS (1998). Characterization of mannose receptor-dependent phagocytosis mediated by Mycobacterium tuberculosis lipoarabinomannan.. Infect Immun.

[22] Brightbill HD, Libraty DH, Krutzik SR, Yang RB, Belisle JT (1999). Host defense mechanisms triggered by microbial lipoproteins through toll-like receptors.. Science.

[23] Heldwein KA, Fenton MJ (2002). The role of Toll-like receptors in immunity against mycobacterial infection.. Microbes Infect.

[24] Astarie-Dequeker C, N'Diaye EN, Le Cabec V, Rittig MG, Prandi J (1999). The mannose receptor mediates uptake of pathogenic and nonpathogenic mycobacteria and bypasses bactericidal responses in human macrophages.. Infect Immun.

[25] Kang PB, Azad AK, Torrelles JB, Kaufman TM, Beharka A (2005). The human macrophage mannose receptor directs Mycobacterium tuberculosis lipoarabinomannan-mediated phagosome biogenesis.. J Exp Med.

[26] Zimmerli S, Edwards S, Ernst JD (1996). Selective receptor blockade during phagocytosis does not alter the survival and growth of Mycobacterium tuberculosis in human macrophages.. Am J Respir Cell Mol Biol.

[27] Ernst JD (1998). Macrophage receptors for Mycobacterium tuberculosis.. Infect Immun.

[28] Tailleux L, Schwartz O, Herrmann JL, Pivert E, Jackson M (2003). DC-SIGN is the major Mycobacterium tuberculosis receptor on human dendritic cells.. J Exp Med.

[29] Gilleron M, Nigou J, Nicolle D, Quesniaux V, Puzo G (2006). The acylation state of mycobacterial lipomannans modulates innate immunity response through toll-like receptor 2.. Chem Biol.

[30] Gilleron M, Quesniaux VF, Puzo G (2003). Acylation state of the phosphatidyl inositol hexamannosides from mycobacterium bovis BCG and mycobacterium tuberculosis H37Rv and its implication in TLR response.. J Biol Chem.

[31] Torrelles JB, Azad AK, Schlesinger LS (2006). Fine discrimination in the recognition of individual species of phosphatidyl-myo-inositol mannosides from Mycobacterium tuberculosis by C-type lectin pattern recognition receptors.. J Immunol.

[32] Eisenbarth SC, Piggott DA, Huleatt JW, Visintin I, Herrick CA (2002). Lipopolysaccharide-enhanced, toll-like receptor 4-dependent T helper cell type 2 responses to inhaled antigen.. J Exp Med.

[33] Sayers I, Severn W, Scanga CB, Hudson J, Le Gros G (2004). Suppression of allergic airway disease using mycobacterial lipoglycans.. J Allergy Clin Immunol.

[34] Ainge GD, Hudson J, Larsen DS, Painter GF, Gill GS (2006). Phosphatidylinositol mannosides: synthesis and suppression of allergic airway disease.. Bioorg Med Chem.

[35] Harper JL, C.L. H, Larsen DS, Painter GF, Gill GS (2010). A PIM2 analogue suppresses allergic airway disease.. Bioorg Med Chem.

[36] Ilangumaran S, Arni S, Poincelet M, Theler JM, Brennan PJ (1995). Integration of mycobacterial lipoarabinomannans into glycosylphosphatidylinositol-rich domains of lymphomonocytic cell plasma membranes.. J Immunol.

[37] Shabaana AK, Kulangara K, Semac I, Parel Y, Ilangumaran S (2005). Mycobacterial lipoarabinomannans modulate cytokine production in human T helper cells by interfering with raft/microdomain signalling.. Cell Mol Life Sci.

[38] Shimazu R, Akashi S, Ogata H, Nagai Y, Fukudome K (1999). MD-2, a molecule that confers lipopolysaccharide responsiveness on Toll-like receptor 4.. J Exp Med.

[39] Nagai Y, Akashi S, Nagafuku M, Ogata M, Iwakura Y (2002). Essential role of MD-2 in LPS responsiveness and TLR4 distribution.. Nat Immunol.

[40] Ohnishi T, Muroi M, Tanamoto K (2003). MD-2 is necessary for the toll-like receptor 4 protein to undergo glycosylation essential for its translocation to the cell surface.. Clin Diagn Lab Immunol.

[41] Huber M, Kalis C, Keck S, Jiang Z, Georgel P (2006). R-form LPS, the master key to the activation ofTLR4/MD-2-positive cells.. Eur J Immunol.

[42] Jiang Z, Georgel P, Du X, Shamel L, Sovath S (2005). CD14 is required for MyD88-independent LPS signaling.. Nat Immunol.

[43] Michelsen KS, Aicher A, Mohaupt M, Hartung T, Dimmeler S (2001). The role of toll-like receptors (TLRs) in bacteria-induced maturation of murine dendritic cells (DCS). Peptidoglycan and lipoteichoic acid are inducers of DC maturation and require TLR2.. J Biol Chem.

[44] Hoshino K, Takeuchi O, Kawai T, Sanjo H, Ogawa T (1999). Cutting edge: Toll-like receptor 4 (TLR4)-deficient mice are hyporesponsive to lipopolysaccharide: evidence for TLR4 as the Lps gene product.. J Immunol.

[45] Moore KJ, Andersson LP, Ingalls RR, Monks BG, Li R (2000). Divergent response to LPS and bacteria in CD14-deficient murine macrophages.. J Immunol.

[46] Jack RS, Fan X, Bernheiden M, Rune G, Ehlers M (1997). Lipopolysaccharide-binding protein is required to combat a murine gram-negative bacterial infection.. Nature.

[47] Riviere M, Moisand A, Lopez A, Puzo G (2004). Highly ordered supra-molecular organization of the mycobacterial lipoarabinomannans in solution. Evidence of a relationship between supra-molecular organization and biological activity.. J Mol Biol.

[48] Kawasaki K, Akashi S, Shimazu R, Yoshida T, Miyake K (2000). Mouse toll-like receptor 4.MD-2 complex mediates lipopolysaccharide-mimetic signal transduction by Taxol.. J Biol Chem.

[49] Byrd-Leifer CA, Block EF, Takeda K, Akira S, Ding A (2001). The role of MyD88 and TLR4 in the LPS-mimetic activity of Taxol.. Eur J Immunol.

[50] Elass E, Coddeville B, Guerardel Y, Kremer L, Maes E (2007). Identification by surface plasmon resonance of the mycobacterial lipomannan and lipoarabinomannan domains involved in binding to CD14 and LPS-binding protein.. FEBS Lett.

[51] Akashi S, Saitoh S, Wakabayashi Y, Kikuchi T, Takamura N (2003). Lipopolysaccharide interaction with cell surface Toll-like receptor 4-MD-2: higher affinity than that with MD-2 or CD14.. J Exp Med.

[52] Hailman E, Lichenstein HS, Wurfel MM, Miller DS, Johnson DA (1994). Lipopolysaccharide (LPS)-binding protein accelerates the binding of LPS to CD14.. J Exp Med.

[53] Janot L, Secher T, Torres D, Maillet I, Pfeilschifter J (2008). CD14 works with toll-like receptor 2 to contribute to recognition and control of Listeria monocytogenes infection.. J Infect Dis.

[54] Pugin J, Heumann ID, Tomasz A, Kravchenko VV, Akamatsu Y (1994). CD14 is a pattern recognition receptor.. Immunity.

[55] Perera PY, Vogel SN, Detore GR, Haziot A, Goyert SM (1997). CD14-dependent and CD14-independent signaling pathways in murine macrophages from normal and CD14 knockout mice stimulated with lipopolysaccharide or taxol.. J Immunol.

[56] Rosenberger CM, Finlay BB (2003). Phagocyte sabotage: disruption of macrophage signalling by bacterial pathogens.. Nat Rev Mol Cell Biol.

[57] Diacovich L, Gorvel JP (2010). Bacterial manipulation of innate immunity to promote infection.. Nat Rev Microbiol.

[58] Hoppe HC, de Wet BJ, Cywes C, Daffe M, Ehlers MR (1997). Identification of phosphatidylinositol mannoside as a mycobacterial adhesin mediating both direct and opsonic binding to nonphagocytic mammalian cells.. Infect Immun.

[59] Fischer K, Scotet E, Niemeyer M, Koebernick H, Zerrahn J (2004). Mycobacterial phosphatidylinositol mannoside is a natural antigen for CD1d-restricted T cells.. Proc Natl Acad Sci U S A.

[60] Zajonc DM, Ainge GD, Painter GF, Severn WB, Wilson IA (2006). Structural characterization of mycobacterial phosphatidylinositol mannoside binding to mouse CD1d.. J Immunol.

[61] Manukyan M, Triantafilou K, Triantafilou M, Mackie A, Nilsen N (2005). Binding of lipopeptide to CD14 induces physical proximity of CD14, TLR2 and TLR1.. Eur J Immunol.

[62] Vignal C, Guerardel Y, Kremer L, Masson M, Legrand D (2003). Lipomannans, but not lipoarabinomannans, purified from Mycobacterium chelonae and Mycobacterium kansasii induce TNF-alpha and IL-8 secretion by a CD14-toll-like receptor 2-dependent mechanism.. J Immunol.

[63] Youn JH, Oh YJ, Kim ES, Choi JE, Shin JS (2008). High mobility group box 1 protein binding to lipopolysaccharide facilitates transfer of lipopolysaccharide to CD14 and enhances lipopolysaccharide-mediated TNF-alpha production in human monocytes.. J Immunol.

[64] Lichtman SN, Wang J, Lemasters JJ (1998). LPS receptor CD14 participates in release of TNF-alpha in RAW 264.7 and peritoneal cells but not in kupffer cells.. Am J Physiol.

[65] Perera PY, Mayadas TN, Takeuchi O, Akira S, Zaks-Zilberman M (2001). CD11b/CD18 acts in concert with CD14 and Toll-like receptor (TLR) 4 to elicit full lipopolysaccharide and taxol-inducible gene expression.. J Immunol.

[66] Trinchieri G (2003). Interleukin-12 and the regulation of innate resistance and adaptive immunity.. Nat Rev Immunol.

[67] Trinchieri G, Sher A (2007). Cooperation of Toll-like receptor signals in innate immune defence.. Nat Rev Immunol.

