# Supplementary figures and images for: Mycobacterial PIMs Inhibit Host Inflammatory Responses through CD14-Dependent and CD14-Independent Mechanisms
Source: PLoS One. 2011 Sep 16;6(9):e24631. doi: 10.1371/journal.pone.0024631 (PMC3174970; doi:10.1371/journal.pone.0024631)

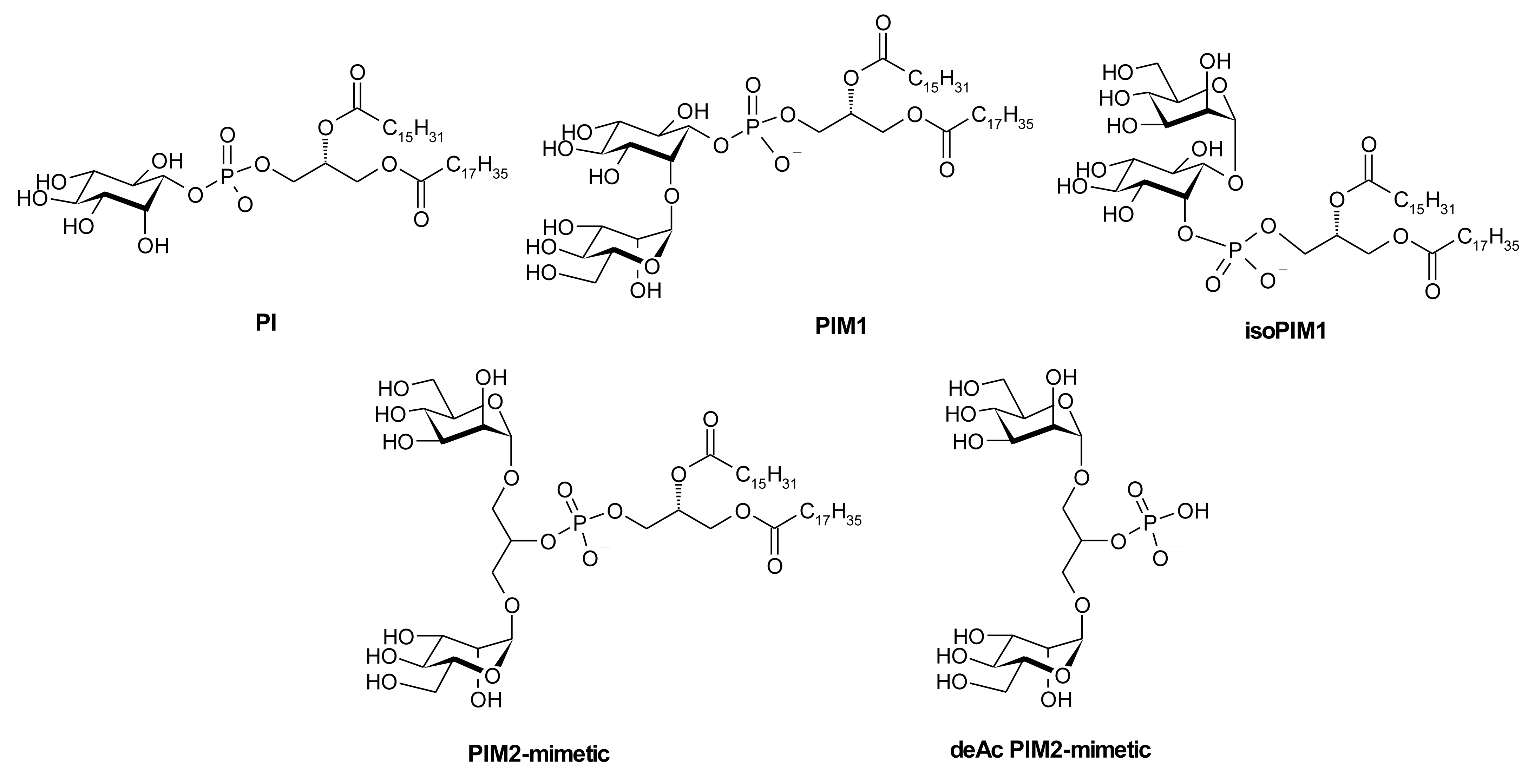

Supplement: Figure S1 — Structure of synthetic PIM1, isoPIM1 and PIM2 mimetics. Schematic representation of synthetic PIM1 showing the C16 and C18 acyl groups on glycerol chain positions sn-2 and sn-1, an isomer of PIM1 (isoPIM1) carrying the phosphatidyl group at position O-2 and the mannosyl residue at O-1 of D-myo-inositol, the precursor PI, a synthetic mimetic of PIM2 (PIM2 mimetic) bearing C16 and C18 acyl chains, and the de-acylated precursor of the PIM2 mimetic (deAcPIM2 mimetic) as control molecule. (TIF) [file pone.0024631.s001.tif]

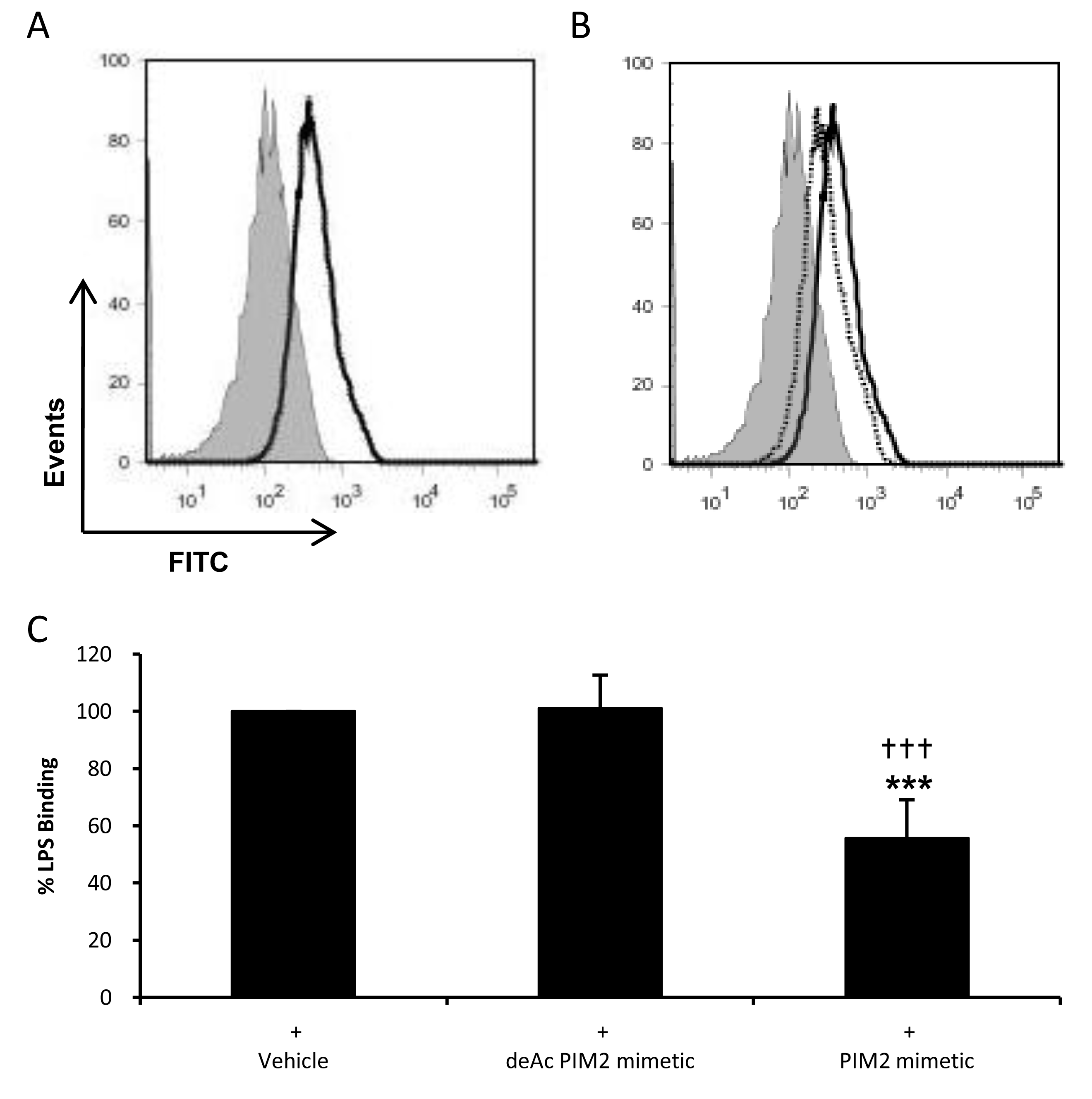

Supplement: Figure S2 — Synthetic PIM analogues inhibit S-LPS-binding to macrophages. Bone marrow derived macrophages from C57Bl/6 mice were incubated with 10 µg/mL (dotted line) deAcPIM2 mimetic (A) or PIM2 mimetic (B) prior incubation with 5 µg/mL of biotinylated S-LPS and streptavidine FITC (black line). DeAcPIM2 mimetic did not displace S-LPS and was superimposed with S-LPS plus vehicle (A). In controls, macrophages were stained only with streptavidin FITC (grey histogram). Results shown are from cells derived from one mouse representative of cells from four different mice. (C) Percentage of S-LPS binding to macrophages in presence of PIMs or vehicle. Mean +/− SD from n = 4 mice from 2 independent experiments. ***, p<0.001 versus vehicle. †††, p<0.001 indicate significant differences between deAcPIM2 mimetic and PIM2 mimetic. (TIF) [file pone.0024631.s002.tif]

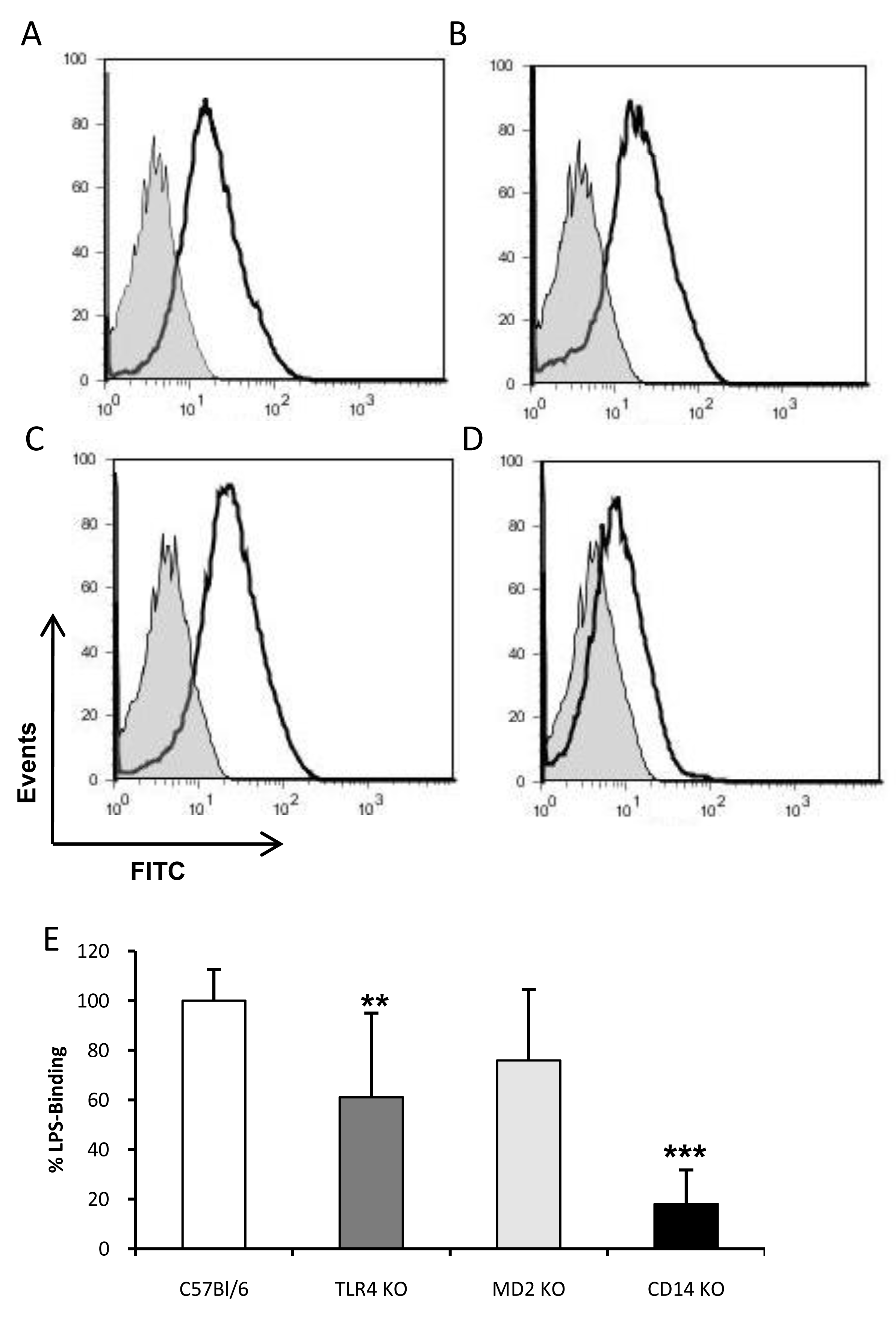

Supplement: Figure S3 — CD14 is an important co-receptor for S-LPS-binding to macrophages. Bone marrow derived macrophages from C57Bl/6 (A), TLR4 KO (B), MD2 KO (C) or CD14 KO (D) mice were incubated with biotinylated S-LPS and streptavidine FITC (black line). In controls, macrophages were only incubated with streptavidine FITC (grey histogram). Results are from one mouse representative of four mice. (E) Percentage of S-LPS-binding to macrophages compared to C57Bl/6 binding level. Mean +/− SD from n = 4–8 mice from two to four independent experiments. **, p<0.01, ***, p<0.001 versus C57Bl/6. (TIF) [file pone.0024631.s003.tif]

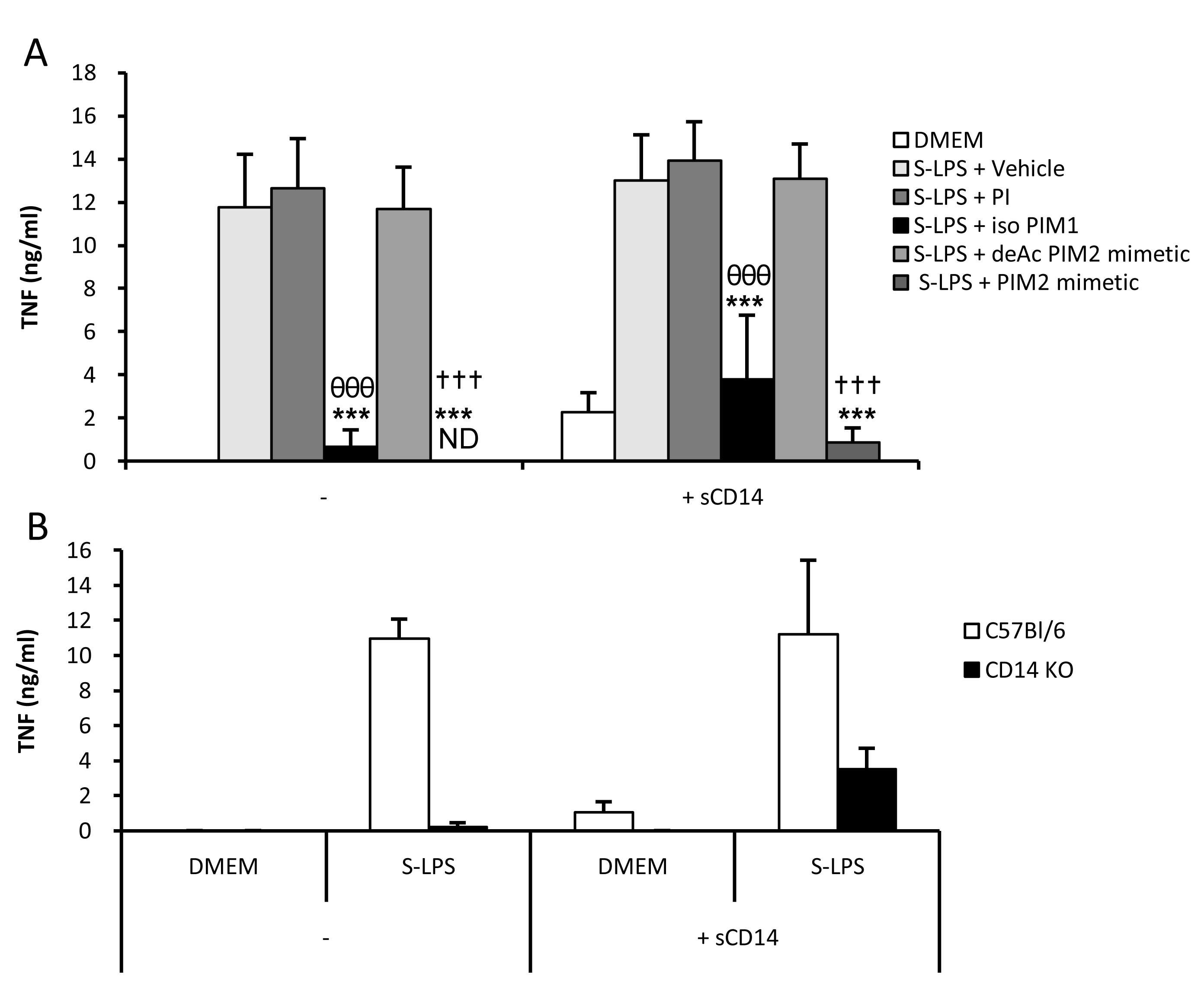

Supplement: Figure S4 — Addition of sCD14 does not affect PIM inhibition of S-LPS-induced TNF. (A) Macrophages from C57Bl/6 mice were incubated with murine soluble CD14 (sCD14; 5 µg/mL) and PIMs (10 µg/mL) as indicated prior to stimulation with S-LPS (100 ng/mL). (B) Wild type or CD14 KO macrophages were stimulated with S-LPS in the absence or in the presence of murine soluble CD14 (sCD14; 5 µg/mL). TNF concentration was measured in the supernatants after overnight incubation. Mean +/− SD from n = 4 mice from two experiments representative of three independent experiments. ***, p<0.001 versus vehicle. θθθ, p<0.001 indicate significant differences between isoPIM1 versus PI as control, †††, p<0.001 indicate significant differences between deAcPIM2 mimetic and PIM2 mimetic. (TIF) [file pone.0024631.s004.tif]

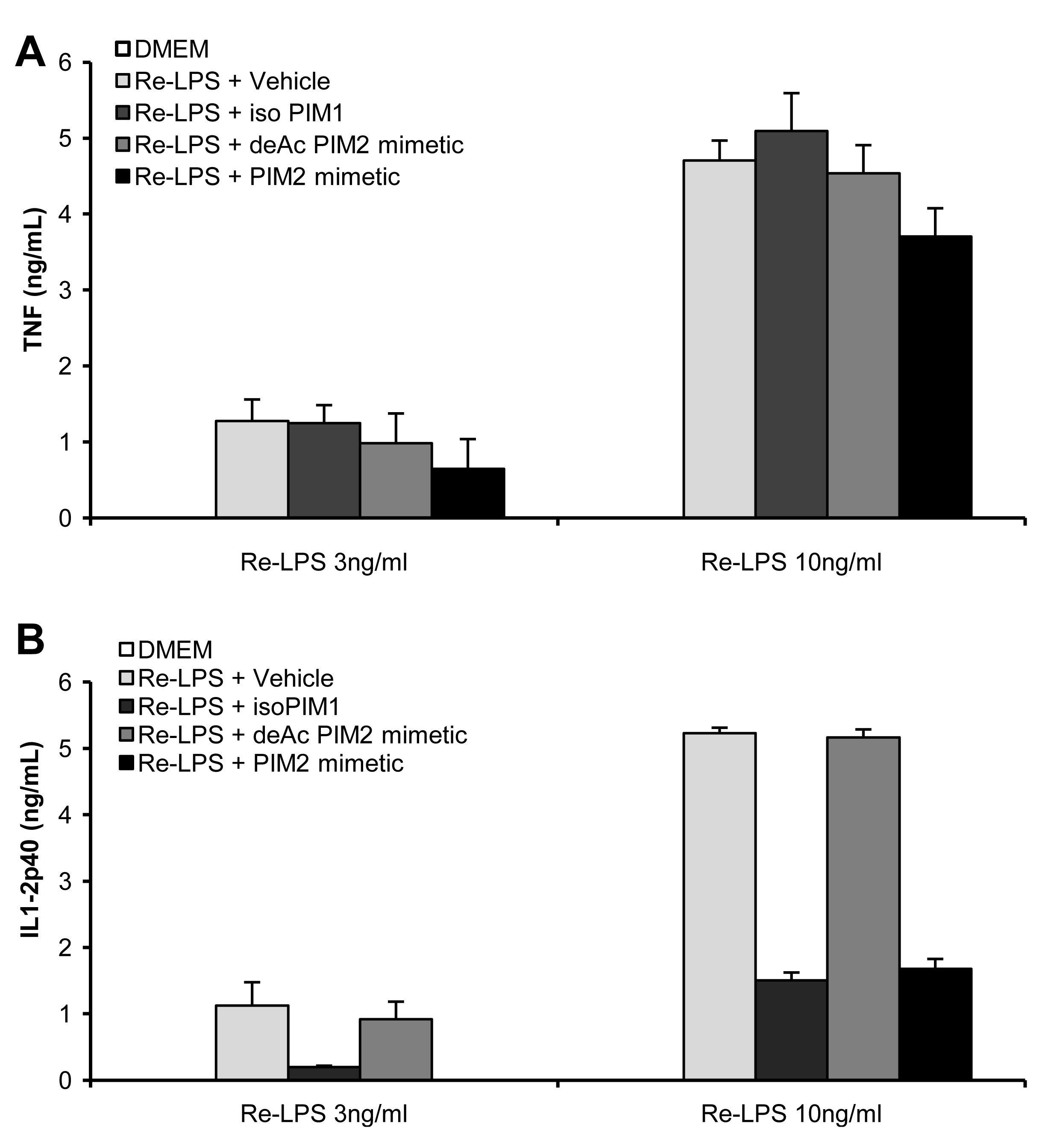

Supplement: Figure S5 — Differential inhibition of induced TNF and IL-12 p40 release by PIMs at low doses of Re-LPS. Concentrations of TNF (A) and IL-12 p40 (B) in supernatants of CD14-deficient macrophages stimulated overnight with 3 or 10 ng/mL of Re-LPS in the presence of synthetic isoPIM1, deAcPIM2 mimetic, PIM2 mimetic (10 µg/mL), or vehicle. Results are mean +/− SD from n = 2 mice. (TIF) [file pone.0024631.s005.tif]

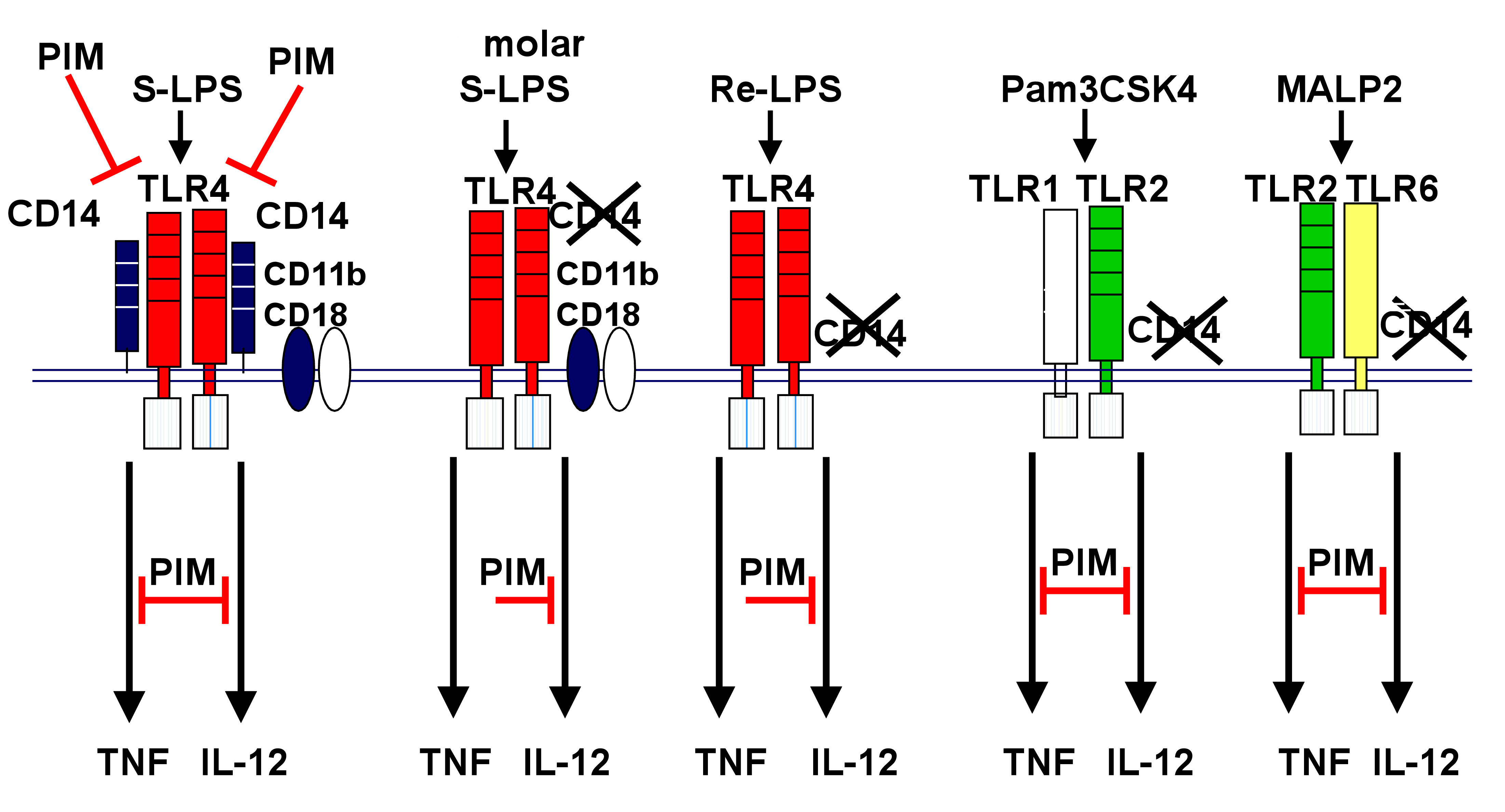

Supplement: Figure S6 — Schematic model of PIM interference with TLR2 and TLR4 responses. PIMs block LPS binding to CD14, which may explain the inhibition of PIM in CD14-dependent LPS functional responses through TLR4. However, not all TLR responses need CD14, as indicated for TLR4 response to rough LPS or to high micromolar doses of smooth LPS, but also for TLR2/TLR1 response to Pam3CSK4 and TLR2/TLR6 response to Malp2. In these cases, PIM inhibitory effect may be downstream of TLRs. In addition, IL-12p40 expression requires other surface molecules to be complete, such as CD11b and CD18, and this may in part explain the different sensitivity of TNF and IL-12p40 to the inhibition by PIMs. (TIF) [file pone.0024631.s006.tif]
